# Geodiversity and resilience: A scoping review

**DOI:** 10.1007/s13280-026-02409-8

**Published:** 2026-05-03

**Authors:** Maija Toivanen, Daniel Santos, Aleksi Räsänen

**Affiliations:** 1 Geography Research Unit, University of Oulu, P.O. BOX 8000, 90014 Oulu, Finland; 2 Centre for Research Support on Geological Heritage and Geotourism (GeoHereditas), Institute of Geosciences, University of São Paulo, São Paulo, 05508-080 Brazil

**Keywords:** Geodiversity, Geoheritage, Resilience, Social-ecological systems, Scoping review

## Abstract

Resilience thinking has become central to addressing environmental and societal challenges, yet it focuses primarily on ecological and social dimensions while physical foundations remain underrepresented. This systematic scoping review examined 90 geodiversity and geoheritage studies (2012–2025) analysing connections to resilience concepts. While most reviewed studies lack explicit resilience frameworks, they demonstrate extensive implicit resilience engagement, particularly through maintaining diversity and redundancy, managing slow variables, encouraging learning, and broadening participation. Geodiversity enriches resilience thinking by treating physical environments not as passive backdrops but as active participants in system change, and by bridging natural and social dimensions that are typically managed separately. Three interrelated barriers limit integration of resilience and geodiversity: disciplinary communities remain disconnected, evidence emphasizes description over mechanisms, and institutional infrastructure for geodiversity governance lags behind that for biodiversity. Overcoming these barriers through collaborative efforts could ground resilience thinking in the geological reality that underlies all sustainability challenges.

**Supplementary Information:**

The online version contains supplementary material available at https://doi.org/10.1007/s13280-026-02409-8.

## Introduction

Resilience has become a central concept in addressing global environmental and societal challenges, from climate change and biodiversity loss to land degradation and natural hazards (Folke 2016). Since Holling’s (1973) foundational work, resilience has evolved across multiple disciplines—from ecological persistence to engineering recovery to social resilience—yet largely within disciplinary silos (Baggio et al. 2015).

Social-ecological resilience research has developed resilience thinking into a more integrative framework that recognizes human–environment interconnections and focuses on capacities to persist, adapt, and transform while maintaining ecosystem health and human well-being (Folke 2016; Folke et al. 2016). However, despite growing recognition that Earth system processes are fundamental to sustainability and resilience (Steffen et al. 2015; Folke et al. 2016; Pascual et al. 2023), the physical foundations upon which ecosystems and societies are built remain underrepresented in resilience thinking.

This gap stems partly from equating nature primarily with biodiversity, overlooking the abiotic diversity that forms the physical template for life and society (Justice et al. 2025). Geodiversity and geoheritage research (see Box 1 for key terminology) offer perspectives to fill this gap, with their holistic understanding of Earth features and processes, recognition of biotic–abiotic and human–nature interdependencies, and attention to dynamic physical foundations and their cultural meanings. Though geodiversity underpins many resilience-related processes, these connections have not been systematically explored. Therefore, we lack a systematic understanding of how geodiversity connects to resilience thinking, what conceptual and practical bridges exist, and how these insights might strengthen existing frameworks spanning social, engineering, ecological, and social-ecological resilience alike.

**Table:** **Box 1** Geodiversity and geoheritage: Concepts and scopes

Together, geodiversity and geoheritage serve as the primary terms for the broader geodiversity research domain. As the abiotic counterpart to biodiversity, geodiversity describes the physical variety of abiotic nature, while geoheritage forms the geological dimension of natural heritage, recognizing specific elements of that variety for their cultural, scientific, or educational significance.
*Geodiversity* describes the natural range of geological, geomorphological, soil, and hydrological features, including their assemblages, structures, systems, and contributions to landscapes (Gray 2013). Developed as a conceptual counterpart to biodiversity since the late 1980s, the concept emerged from efforts to protect geological sites but has evolved towards broader recognition of how abiotic features underpin ecosystem functioning and human well-being (Gray 2023). Geodiversity research has expanded to encompass developing assessment methods and quantification approaches, as well as examining connections with biodiversity, ecosystem processes, and human systems (Kubalíková et al. 2023).
*Geoheritage* recognizes specific geological features for their significance to conservation and society, serving as geodiversity’s cultural counterpart (Gray 2025). Definitions range from emphasizing scientific importance to encompassing broader scientific, educational, cultural, aesthetic, and economic values (Brilha 2025). Research ranges from site identification, inventories, and protection to applied work in geotourism and geoeducation, with the field increasingly becoming more multidisciplinary beyond these traditional themes (Kubalíková et al. 2023).
More broadly, geodiversity literature has developed a family of *geo*-prefixed concepts—including *geoconservation* (the practice of protecting and managing geodiversity and geoheritage) and *geosystem services* (the benefits geodiversity provides to ecosystems and societies)—that bring the non-living dimensions of nature into conversations long dominated by biodiversity.

**Fig. 1:**
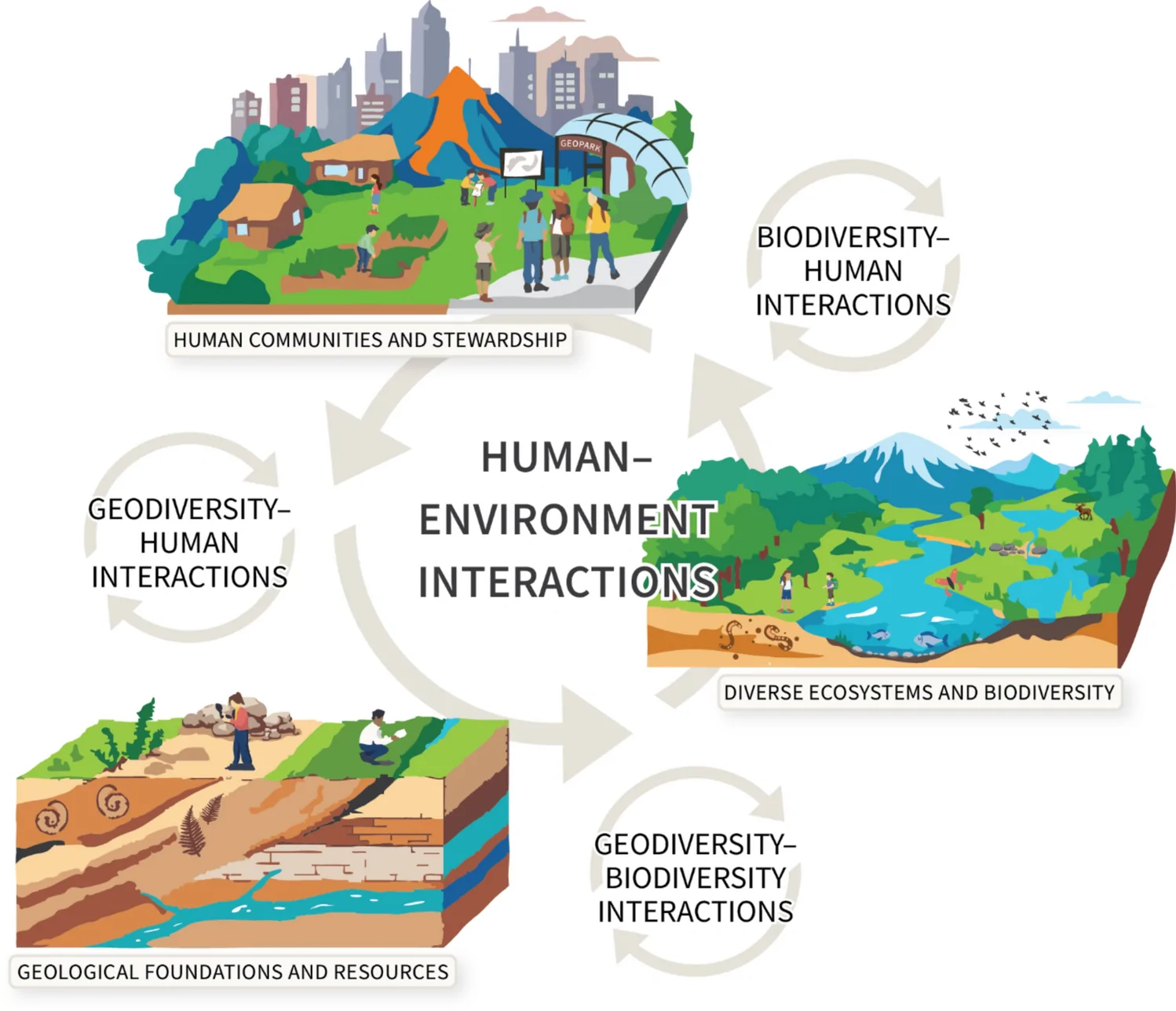
Human–environment interactions involve more than the relationship between people and living nature. Geological foundations, its rocks, landforms, soils, waters, forms the physical foundation of ecosystems and societies, actively shapes how they develop and change, and provides resources for both nature and people. These contributions, both independently and through interactions with biodiversity, are part of what makes human–environment relationships whole

This gap has structural roots. Biodiversity emerged from the 1992 Rio Earth Summit as a shared language enabling researchers, practitioners, and policymakers to recognize, assess, and protect the variety of life, backed by international conventions, monitoring frameworks, and funding mechanisms. Geodiversity, though proposed as a parallel integrative concept for abiotic nature around the same time, did not gain similar institutional support (Gray 2021). Research on Earth’s physical variety has instead developed across fragmented disciplinary communities (e.g. geology, pedology, geomorphology, hydrology, geoconservation) without shared language or institutional infrastructure. Geodiversity and geoheritage research have emerged partly in response to this fragmentation, seeking to build an integrative framework for understanding human–environment interactions that gives abiotic nature equal standing alongside biodiversity (Fig. 1). In doing so, it explicitly challenges the biocentric assumptions embedded also in sustainability and resilience sciences. The bias runs deep enough to be encoded in the terminology of resilience and sustainability sciences, where the dominant framework is called “social-ecological” rather than “social-environmental”, implicitly centring living nature. The gap is therefore not primarily about missing knowledge, but about missing integration.

This review addresses that integration gap by systematically examining how geodiversity and geoheritage relate to resilience. We address two research questions: RQ1) How is resilience conceptualized and applied in geodiversity and geoheritage literature and RQ2) What are the key concepts and mechanisms through which geodiversity is linked to resilience.

## Materials and methods

### Search strategy and literature screening

**Fig. 2:**
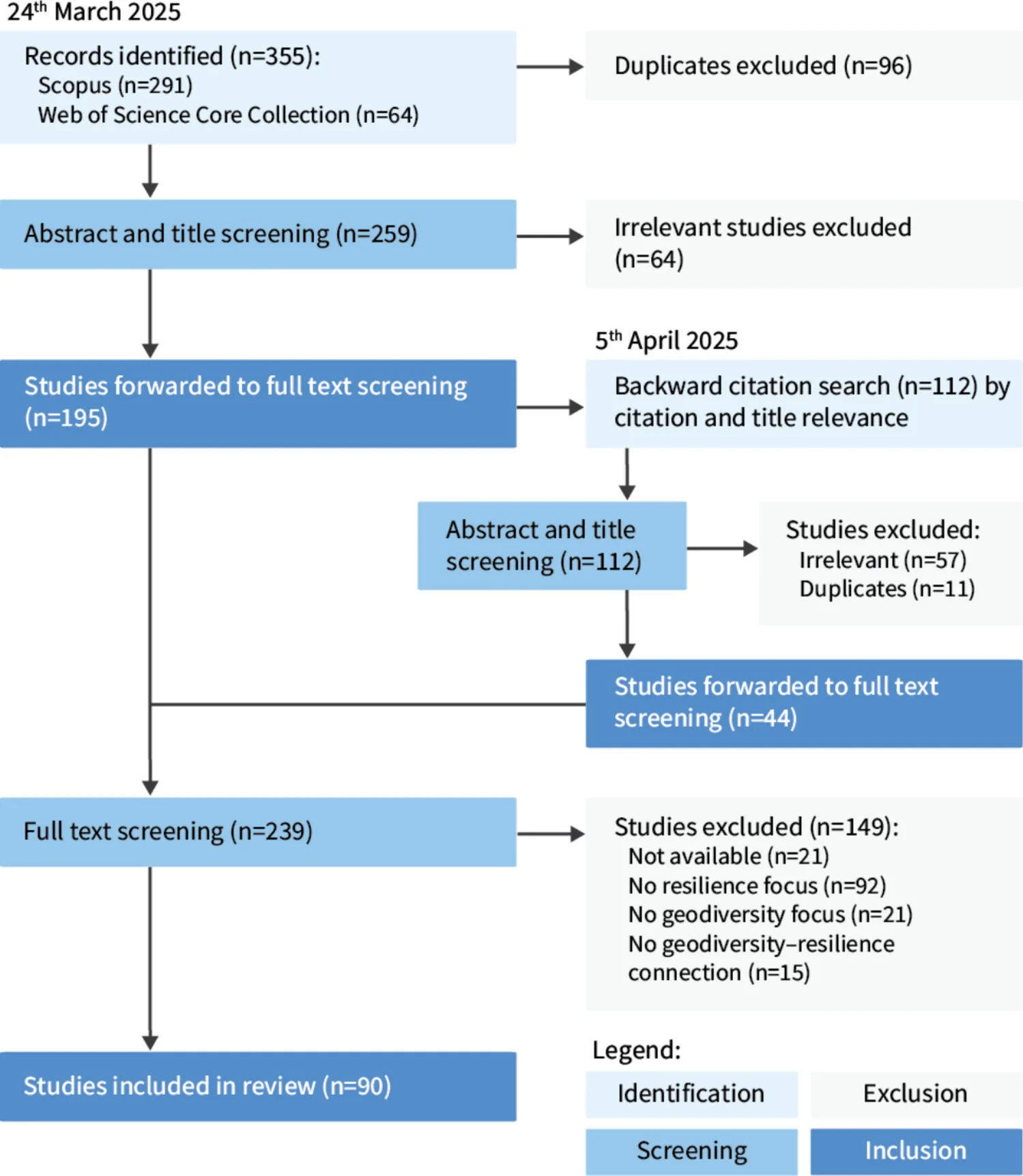
PRISMA flowchart for systematic literature search and screening

We conducted a systematic scoping review following PRISMA scoping review guidelines (Tricco et al. 2018) using Covidence software for screening and data management (Fig. 2). A systematic scoping review was chosen given the heterogeneous terminology and emerging nature of this research area.

We searched Scopus and Web of Science Core Collection in March 2025. The search strategy combined terms for geodiversity and resilience using the following strings: (“geodiversity” AND “resilience”) and (“geoheritage” AND “resilience”), where “geodiversity” and “geoheritage” were limited to title, abstract, and keywords, and “resilience” was searched across all fields. This approach ensured focus on geodiversity-centred research while capturing mentions of resilience in metadata, such as cited references.

To address geodiversity terminology inconsistencies, we conducted backward citation screening of studies forwarded to full-text screening. Rather than following a fixed protocol, this screening relied on the screener’s expertise in geodiversity and related earth science terminology to identify relevant studies that may not have used “geodiversity” in their title/abstract/keywords. We identified additional studies based on title relevance to geodiversity or related themes (e.g. geological features, soils, landforms, geotourism) and resilience, with particular attention to references cited in sections where geodiversity–resilience connections were discussed. This context-dependent approach was intentional, allowing us to capture conceptually relevant literature across the fragmented literature.

Studies were included if they addressed geodiversity and made explicit or implicit connections to resilience. We included all study designs, publication types, and geographic contexts to capture the breadth of this emerging research area. Studies were excluded if they lacked substantive focus on either geodiversity or resilience, mentioned both concepts without connecting them, or were inaccessible despite attempts through institutional access and author contact. All included studies were in English, with no date restrictions applied. Full-text screening resulted in 90 studies meeting inclusion criteria.

### Data extraction and analysis

We developed a structured data analysis template (Table S1) to systematically extract: (1) general publication characteristics, (2) geodiversity focus, (3) resilience conceptualization, (4) geodiversity–resilience relationships, and (5) connections to the seven principles of resilience. One reviewer (author MT) extracted data using an iterative process. After pilot testing on ~40% of studies, the template was refined to better capture how resilience was addressed, shifting from categorical coding to more descriptive observations. Previously extracted data were re-analysed using the updated template for consistency.

In the analysis, we first characterized the literature through descriptive analysis of publication trends, geographic distribution, study designs, and disciplinary contexts. We then conducted analysis structured around our two research questions.

**For RQ1** (*How is resilience conceptualized and applied in geodiversity and geoheritage literature?*), we documented how resilience was conceptualized across its full disciplinary range (social, engineering, ecological, and social-ecological) without restricting extraction to any single definition, since capturing this variation was itself a research objective. We assessed the level of engagement (explicit, implicit, or mentioned only), noting whether studies provided formal definitions, applied established frameworks, or demonstrated understanding through context.

**For RQ2** (*What are the key concepts and mechanisms through which geodiversity is linked to resilience?*), we mapped study findings against the seven principles for building resilience in social-ecological systems (Biggs et al. 2012, 2015; Table 1). This widely used framework (Baird et al. 2024) organizes resilience-building strategies into principles concerning what to manage (system diversity, connectivity, and slow variables; P1–P3) and principles concerning how to manage (through adaptive thinking, learning, participation, and governance; P4–P7). We selected it because it bridges environmental and social dimensions and provides a common language to facilitate dialogue across research communities. We applied the principles to all reviewed studies regardless of how they framed resilience, allowing us to identify geodiversity’s contributions to resilience mechanisms even in studies that did not explicitly engage with social-ecological resilience theory. We characterized how geodiversity is portrayed in relation to resilience (as facilitator, constraint, or mixed), identified types and quality of evidence presented, and documented the spatial scales at which connections operated.

**Table 1:** The seven principles for building resilience in social-ecological systems (Biggs et al. 2012, 2015), grouped by their focus on what to manage (P1–P3) and how to manage (P4–P7), with key messages summarized

Category	Abbreviation	Principle	Resilience principle key message
What to manage	P1	Maintain diversity and redundancy	Multi-component systems are more resilient than simpler systems, as redundancy provides insurance if parts fail
	P2	Manage connectivity	Systems with high connectivity can recover from shocks quickly but too-connected systems can enable rapid spread of harm
	P3	Manage slow variables and feedbacks	Slow variables and feedbacks are crucial for the systems to persist and function within their current state or development trajectory
How to manage	P4	Foster complex adaptive systems thinking	Social-ecological (or social-environmental) systems are built upon complex and unpredictable interdependencies which need to be understood to the system to be managed and governed
	P5	Encourage learning	Learning and experimentation safeguards that various knowledge types are accounted for in system management and governance
	P6	Broaden participation	Participation of a wide variety of actors builds trust and enables constructing shared and versatile understanding of the system and its components
	P7	Promote polycentric governance systems	Well-connected governance structures with various collaborative actors enable dealing with changes and disturbances in a timely and just manner

### Methodological limitations

Several methodological limitations should be considered. First, the search strategy was deliberately focussed on geodiversity and geoheritage as marker keywords. Pilot searches adding related terms (e.g. geoconservation, geosystem services) yielded no additional results, confirming these terms effectively capture the relevant literature. Correspondingly, studies on abiotic processes (e.g. geology, geomorphology, soils, hydrology) that do not use geodiversity terminology fall outside our scope, because this review focuses on how the geodiversity concept is connected to resilience, not on abiotic diversity as such. Studies lacking this terminology are unlikely to engage with the conceptual framework under review. Second, single-researcher data extraction introduces potential for subjective interpretation. To manage this, we developed and refined the data analysis template through discussion among the authors and re-analysed previously extracted data following template updates for consistency. Third, restricting the search to Scopus and Web of Science Core Collection may have missed especially grey literature, but these databases provide strong coverage of relevant fields in which geodiversity–resilience connections are discussed, as reflected in the 42 journals represented in our final sample. Backward citation screening further compensated for potential gaps. Fourth, limiting searches to English-language articles may have excluded relevant work in other languages. Finally, no review protocol was prospectively registered, which we acknowledge as a limitation, but a completed PRISMA-ScR checklist is provided as Table S2.

## Results

### Overview of the reviewed literature

We reviewed 90 studies (Table S3) published between 2012 and 2025, with most (n = 63) appearing after 2020. Studies were predominantly empirical (n = 51), followed by theoretical and conceptual papers (n = 16), reviews (n = 14), methodological contributions (n = 5), and editorials or position papers (n = 4). Geographic coverage was global, with studies concentrated in Europe (n = 29), Asia (n = 15), and North America (n = 11), and fewer from Africa (n = 4), South America (n = 6), and Pacific islands (n = 3). Additionally, many studies took global or multi-regional perspectives (n = 22). Fourteen studies focussed on geoparks. Research clustered around climate change adaptation and biodiversity conservation, geoheritage assessment and geoconservation, geotourism, and volcanic geohazards. Studies appeared across 42 journals spanning geoheritage, Earth sciences, ecology, and sustainability sciences.

### Patterns of engagement with resilience

The reviewed studies demonstrated variable engagement with resilience theory, from explicit theoretical grounding to implicit application to passing mentions (Table 2).

**Table 2:** Three levels of engagement with resilience concepts in reviewed geodiversity and geoheritage literature (n = 90). See Table S4 for more information on studies with explicit engagement

Level of engagement	Definition
Explicit engagement (n = 17)	Studies that provided formal definitions of resilience with citations to resilience literature, integrated resilience as a central theoretical framework, or operationalized resilience through specific metrics or assessment tools
Implicit engagement (n = 46)	Studies that used resilience terminology throughout their analysis without formal definitions, demonstrated understanding through application and context, and showed conceptual alignment with resilience thinking through methodology or cited literature
Mentioned only (n = 27)	Studies where resilience appeared only in passing, treated as self-evident without substantive engagement, typically as isolated aspirational terms

Seventeen studies **explicitly engaged with resilience concepts** through formal definitions and theoretical grounding (Table S4). Most drew primarily on foundational (social-)ecological resilience literature (Anderson et al. 2014; Knudson et al. 2018; Powell et al. 2018; Petrosino et al. 2019; Roque Guerrero et al. 2020; Moretti et al. 2021; Moradipour et al. 2024; Pijet-Migon and Migon 2024), particularly citing Holling (1973), Walker et al. (2004), and Folke (2006). However, these studies often provided multiple conceptualizations, ranging from system-level capacity to absorb disturbances and maintain function to site-specific operationalizations. Beyond ecological resilience, several studies traced concepts from materials science and engineering (De Pascale et al. 2017; Tavasoli et al. 2022), disaster risk reduction (Vereb et al. 2020a; Alberico et al. 2023), and social resilience (Powell et al. 2018). Studies operationalized resilience at multiple spatial scales, from site-level assessments (Anderson et al. 2014, 2023; Zeller et al. 2024) to landscape and community frameworks (Knudson et al. 2018; Powell et al. 2018; Roque Guerrero et al. 2020).

Few studies developed resilience conceptualizations specific to geodiversity contexts. These included defining “geoheritage resilience” (Moradipour et al. 2024) and “site resilience” (Anderson et al. 2014, 2023), developing frameworks for assessing adaptive capacity of geoheritage (Gordon et al. 2022), and building resilience theory for geoheritage sites (Baitalik 2024). Some studies explicitly distinguished resilience from sustainability (Moretti et al. 2021; Pijet-Migon and Migon 2024), or emphasized their interconnection (Alberico et al. 2023).

Approximately half of the studies (46 out of 90) demonstrated **implicit understanding of resilience**, applying concepts without formal theoretical grounding and commonly framing resilience as adaptive capacity to environmental stressors in natural and social systems.

Resilience appeared most prominently in three contexts: geohazard preparedness (Vereb et al. 2020a; Guilbaud et al. 2021; Németh et al. 2021; Morino et al. 2022; Németh et al. 2023; Vegas et al. 2023; Alfama et al. 2024; Arias et al. 2025), climate change adaptation and biodiversity conservation (Comer et al. 2015; Gordon et al. 2018, Crofts 2019; Gordon et al. 2021), and dryland ecosystems experiencing drought (Stavi et al. 2019, 2021; De Falco et al. 2021; Basson et al. 2023). Studies applied resilience concepts across multiple scales, from individuals (Gordon 2023) and communities (Salhi et al. 2020; Das et al. 2024) to landscapes (Harmon and Viles 2013; Matthews 2014; Pelacani et al. 2024). A subset of studies linked geological knowledge to resilience building suggesting that education about geological processes builds resilience, often mentioning UNESCO IGCP Project 692 “Geoheritage for Geohazard Resilience” (Fepuleai et al. 2021; Kubalíková et al. 2023; Németh et al. 2023).

Notably, despite minimal explicit resilience terminology, some studies demonstrated sophisticated understanding of resilience and complex adaptive systems thinking through their analytical frameworks and methodological approaches (Brazier et al. 2012; Harmon and Viles 2013; Matthews 2014; Everard and Quinn 2015; Stavi et al. 2019).

Twenty-seven studies **mentioned resilience** only in passing, treating it as self-evident without substantive engagement, typically as isolated aspirational terms in abstracts or conclusions. This includes references to resilience-related programmes and policy frameworks (UNESCO IGCP 692, Sendai Framework) where the resilience concept itself was not substantively engaged.

### Nature of the relationship between geodiversity and resilience

Most studies (77 out of 90) portrayed geodiversity as facilitating resilience, while 11 studies showed mixed relationships and two were unclear. No studies portrayed geodiversity purely as constraining resilience.

This predominant pattern of **geodiversity as facilitating resilience** partly reflects the framing of geodiversity literature itself. For example, studies invoking the Conserving Nature’s Stage rationale—the idea that geodiversity provides a stable physical foundation for biodiversity adaptation under environmental change—or ecosystem services logic tend to cast geodiversity as an inherently resilience-enhancing foundation for biodiversity adaptation under environmental change (Gill et al. 2015; Knudson et al. 2018) or for ecosystem functioning and broader services delivery more widely (Gordon et al. 2012; Gordon and Barron 2013; Gray et al. 2013), with its constraints receiving little attention. Similarly, studies examining geohazard contexts or geotourism tend to frame geodiversity as a resource for community preparedness or economic development rather than as a source of constraint (Powell et al. 2018; Paola Petrosino et al. 2019; Agastya et al. 2024; Pijet-Migoń and Migoń 2024).

**The mixed portrayals,** however, recognized geodiversity’s dual nature: volcanic features simultaneously create hazards and provide fertile soils or educational opportunities (Moretti et al. 2021; Alfama et al. 2024), coastal geodiversity offers both advantages and vulnerabilities (from leisure to erosion; Mariani and Melis 2022), and geomorphic processes can limit ecosystem stability while being intrinsic components of geodiversity (Aleksova et al. 2024; Alyaqout and Anzah 2025). Some studies examined geodiversity’s vulnerability to anthropogenic and climate pressures (Kubalíková and Balková 2023; Németh et al. 2023; Moradipour et al. 2024), highlighting it as both source and target for resilience-building.

Reviewed studies addressed multiple dimensions of resilience. *Climate resilience* appeared particularly within the Conserving Nature’s Stage conservation strategy, which positions geodiversity as stable physical foundation for species adaptation (Anderson et al. 2014, Comer et al. 2015; Magness et al. 2018; Alahuhta et al. 2019; Anderson et al. 2023; Zhu et al. 2023). *Ecological resilience* emerged in studies on ecosystem function under stress, especially drought resistance in drylands (Stavi et al. 2019, 2021; De Falco et al. 2021; Basson et al. 2023), and landscape stability (Tavasoli et al. 2022; Gonçalves et al. 2024). *Community resilience* appeared in geopark contexts emphasizing economic diversification and cultural identity (Salhi et al. 2020; Alfama et al. 2024; Pijet-Migon and Migon 2024; Yuliawati et al. 2024). *Geohazard resilience* focussed on disaster risk reduction and hazard education, especially in volcanic or seismic settings (Vereb et al. 2020a; Alberico et al. 2023; Agastya et al. 2024) including visitor safety in hazardous tourism environments (Vereb et al. 2020b). Additional dimensions included *cultural heritage resilience* in hazard-prone areas (Das et al. 2024) and *infrastructure resilience* related to geological resource extraction (Ferrer et al. 2021). In all, geodiversity–resilience connections operated through various physical, ecological, educational, economic, and social mechanisms, and the following analysis is structured around the seven principles of resilience to examine these in more detail.

### Alignment with the seven principles of resilience

Analysis against the seven principles revealed an uneven distribution (Table 3; Table S3). Geodiversity’s contributions were the strongest in principles concerned with physical variety and redundancy (P1, n = 65), temporal dynamics (P3, n = 39), landscape connectivity (P2, n = 29), and education and community engagement through geoheritage (P5, n = 51; P6, n = 37), whereas complex adaptive systems thinking (P4, n = 18) and polycentric governance (P7, n = 4) remain less developed. This asymmetry reflects both the strengths of geodiversity research—particularly its focus on documenting physical variety, temporal depth, and its well-developed research traditions of education and public engagement—and a structural gap in how geodiversity is governed and institutionalized.

**Table 3:** Geodiversity’s contributions to the seven resilience principles (Biggs et al. 2012, 2015), key mechanisms, illustrative examples from the reviewed literature, and suggested applications for conservation and management practice. n = number of reviewed studies (out of 90) coded as connecting to this principle

	Resilience principle	Geodiversity contribution	Example studies	Practical applications
What to manage in systems	P1, Maintain diversity and redundancy (n = 65)	Diverse terrain provides spatial refugia and climate refuges. Varied substrates offer functional alternatives for resources and ecosystem processes. Features changing at different rates provide temporal insurance against disturbance	Brazier et al. (2012), Anderson et al. (2014), Comer et al. (2015), Gill et al. (2015), Knudson et al. (2018), Stavi et al. (2019), Anderson et al. (2023), Basson et al. (2023)	Protect diverse physical settings to maintain adaptation options Recognize geological diversity as foundational to ecological and social resilience
	P2, Manage connectivity (n = 29)	Landscape structure shapes flows of organisms, water, materials, and people. Topography creates beneficial corridors and protective barriers. Physical features influence what spreads and what is contained across landscapes	Brazier et al. (2012), Everard and Quinn (2015), Magness et al. (2018), Stavi et al. (2021), Zacharová et al. (2022), Anderson et al. (2023)	Map physical connectivity patterns Identify landscape features serving as corridors or barriers Optimize connectivity based on what should spread and what should be contained
	P3, Manage slow variables and feedbacks (n = 39)	Physical features change gradually and operate as slow variables controlling system responses. Gradual changes can shift system states before ecological or social impacts become apparent. Geological records reveal past stability limits and threshold crossings, and changes to physical features may be difficult or impossible to reverse	Brazier et al. (2012), Matthews (2014), Gill et al. (2015), Vereb et al. (2020a), Moretti et al. (2021), Gordon et al. (2022), Basson et al. (2023), Gonçalves et al. (2024)	Monitor slow-changing physical features Protect physical features that buffer disturbance Use geological records to identify thresholds Work with natural feedbacks
How to manage systems	P4, Foster complex adaptive systems thinking (n = 18)	Physical features actively shape system dynamics rather than act as passive backdrop. Physical landscapes and human communities mutually shape each other over time. Changes in physical processes cascade through ecological and social systems	Brazier et al. (2012), Gordon et al. (2012), Matthews (2014), Knudson et al. (2018), Moretti et al. (2021), Das et al. (2024)	Adopt integrated physical-ecological-social frameworks Account for uncertainty in physical system responses Match governance scales to physical system dynamics
	P5, Encourage learning (n = 51)	Geological sites provide learning platforms for understanding Earth processes and hazard preparedness. Geological archives extend learning beyond living memory. Geoconservation provides a foundational knowledge framework distinct from but complementary to biodiversity conservation	Gordon and Barron (2013), Gray et al. (2013), Gill et al. (2015), Scarlett and Riede (2019), Petrosino et al. (2019), Németh et al. (2021), Fepuleai et al. (2021), Agastya et al. (2024), Arias et al. (2025)	Monitor contemporary changes and geological archives Engage diverse knowledge holders Establish collaborative platforms (e.g. geoparks) for knowledge sharing
	P6, Broaden participation (n = 37)	Geological features create focal points for community engagement and place attachment. Geoparks involve diverse stakeholders in their management processes. Collaborative governance of geological heritage builds collective capacity	Tavares et al. (2015), Powell et al. (2018), Fepuleai et al. (2021), Németh and Németh (2023), Alfama et al. (2024), Pijet-Migon and Migon (2024)	Connect geological features to community values and livelihoods Involve stakeholders from local to scientific communities Build capacity through training in assessment and management
	P7, Promote polycentric governance systems (n = 4)	Physical features operate across scales that rarely align with administrative boundaries or political timescales. Governing geodiversity requires coordinating multiple interests across local, national, and international levels. Geopark networks exemplify multi-level coordination in practice	Vereb et al. (2020a), Németh et al. (2021), Németh and Németh (2023), Pijet-Migon and Migon (2024)	Build coordination across community, national, and international levels Bridge temporal mismatches between processes and governance cycles Develop institutional infrastructure supporting geodiversity governance

P1’s (*Maintain diversity and redundancy*) dominance reflects the foundational logic of geodiversity research: physical variety functions as insurance through spatial redundancy (species finding suitable habitat as conditions shift; Anderson et al. 2014, 2023), functional redundancy (varied terrain providing multiple water access routes during drought; Stavi et al. 2019; Basson et al. 2023), and response diversity (differential responses to disturbance across geologically varied settings; Brazier et al. 2012; Gill et al. 2015). P2 (*Manage connectivity*) extends this logic from variety to spatial structure, addressing how landscape configuration shapes the flows of organisms, water, and people, from topographic corridors for species movement (Anderson et al. 2023), and geomorphological control of hydrological connectivity (Brazier et al. 2012; Everard and Quinn 2015), to historical agricultural landforms functioning as present-day biodiversity corridors (Zacharová et al. 2022), and geological sites connecting communities around shared landscapes (Morante-Carballo et al. 2023). P3 (*Manage slow variables and feedbacks*) adds a temporal dimension: physical features act as slow variables controlling faster ecological and social processes (Brazier et al. 2012; Matthews 2014; Basson et al. 2023), with the added significance that changes to these features may be difficult or impossible to reverse (Gordon et al. 2022; Moradipour et al. 2024).

P5 (*Encourage learning*) and P6 (*Broaden participation*) are strongly represented, reflecting geoheritage research’s well-developed traditions of education and community engagement. For P5, geological archives extend learning beyond living memory in ways ecological monitoring alone cannot provide (Gordon and Barron 2013; Gray et al. 2013), and geoparks function as educational platforms and natural laboratories for understanding Earth processes and hazard preparedness (Agastya et al. 2024; Arias et al. 2025). For P6, community engagement extends beyond formal participation to place attachment strengthened by geological heritage (Powell et al. 2018) and the integration of indigenous geological knowledge with scientific expertise (Németh and Németh 2023).

The relative low observations of P4 (*Foster complex adaptive systems thinking*) are somewhat paradoxical. Recognizing the interconnected nature of physical, ecological, and social systems is a core motivation of geodiversity research, evident in studies tracing how geological variety shapes biodiversity and human well-being (Brazier et al. 2012; Gordon et al. 2012; Matthews 2014). Yet this recognition rarely translates into the explicit (complex adaptive systems) frameworks that resilience thinking employs, and practical tools for integrating geodiversity into specific management contexts remain mostly absent. The near-absence of P7 (*Promote polycentric governance systems*) is the most evident gap, and its implications and pathways for addressing it are taken up in the discussion.

## Discussion

This review of 90 studies reveals rich connections between geodiversity and resilience across diverse contexts, from climate adaptation to community identity. However, these connections remain largely implicit. While geodiversity and geoheritage research intuitively recognizes its relevance to resilience, most studies lack explicit frameworks articulating why and how these relationships operate. Regarding conceptualization (RQ1), studies demonstrate varied engagement with resilience concepts but rarely connect to established resilience theory. Regarding mechanisms (RQ2), research widely documents geodiversity’s contributions (diverse habitats, educational resources, spaces for participation) but less frequently explains underlying dynamics during disturbances or implications for management.

Although synthesizing insights across contexts, or translating them into integrated management approaches, remains challenging, making these connections explicit offers significant opportunities. Our analysis reveals that geodiversity research already engages extensively with core resilience principles, providing a fruitful foundation for deeper integration. We advance this geodiversity–resilience integration through three steps: examining how geodiversity concepts can enrich resilience thinking (4.1), using UNESCO Global Geoparks as an institutional case study to explore how these connections can be operationalized in practice (4.2), and identifying pathways forward (4.3).

### Enriching resilience thinking with geodiversity and geoheritage

Our review revealed use of multiple resilience framings, from engineering and ecological to social and social-ecological resilience, applied in contexts ranging from climate adaptation and disaster risk reduction to biodiversity conservation and community resilience. While this discussion focuses primarily on social-ecological resilience—or more specifically, social-*environmental* resilience—the heterogeneity reveals geodiversity and geoheritage research’s relevance across complementary resilience dimensions, from ecological (especially prevalent in geodiversity research) to social (especially prevalent in geoheritage research).

**Fig. 3:**
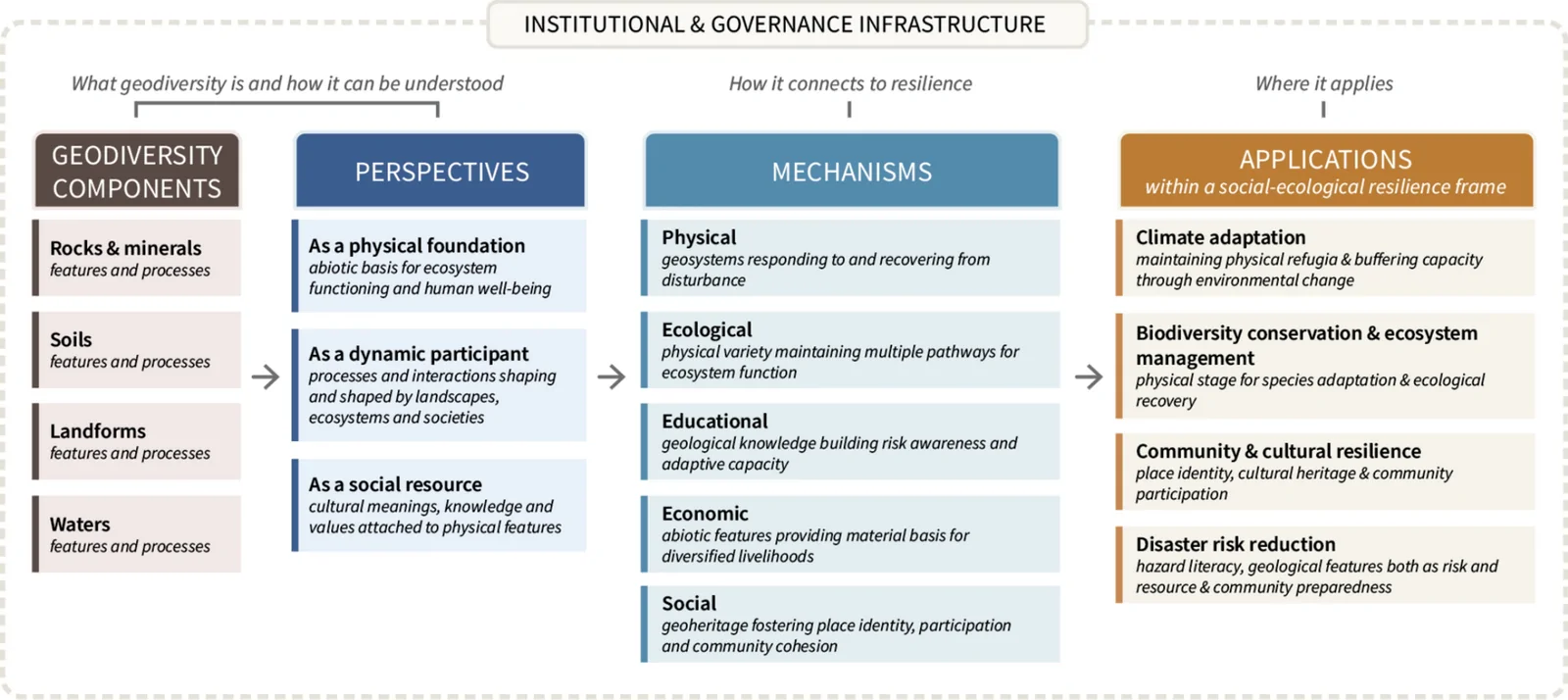
Geodiversity and its components (rocks and minerals, landforms, soils, and waters) can be understood through three perspectives: as a physical foundation, a dynamic participant in how natural and social systems operate, and a social resource. Each perspective reveals mechanisms through which geodiversity contributes to resilience, operating through physical, ecological, educational, economic, and social pathways. Together these mechanisms translate into resilience applications across contexts such as climate adaptation, biodiversity conservation, community resilience, and disaster risk reduction. All connections are mediated by institutional and governance infrastructure (outer frame), which shapes whether and how geodiversity’s contributions are recognized and managed in practice

We identify and discuss three main contributions to resilience thinking: making Earth’s abiotic foundations explicit in what we manage (4.1.1), viewing the physical environment as an active participant in how systems operate (4.1.2), and acknowledging physical features as both natural and social resources (4.1.3). Figure 3 illustrates how these three perspectives connect through physical, ecological, educational, economic, and social mechanisms to resilience applications, mediated by institutional and governance infrastructure.

#### From implicit backdrop to explicit foundation

Ecological resilience research typically frames nature’s contributions through biodiversity and ecosystem services. Since Tansley (1935) formalized the ecosystem concept, research has focussed primarily on living organisms and their interactions, treating physical features as passive backdrop (Pettorelli and Bridgewater 2025). Yet geodiversity actively shapes how ecological and social systems operate, respond to change, and persist over time.

When physical dimensions appear in sustainability frameworks like biosphere stewardship or planetary boundaries (Folke et al. 2021), they typically emphasize selected processes, such as atmospheric composition, biogeochemical cycles, or biodiversity–soil interactions, rather than abiotic diversity itself. For instance, planetary boundaries assess Earth system stability through flows of carbon, water, and nutrients (Lade et al. 2019; Richardson et al. 2023), treating the geophysical environment primarily as medium through which ecological processes operate. What is missing is systematic attention to geological, landform, soil, and hydrological diversity as something requiring management alongside biological diversity.

Geodiversity research addresses this by systematically documenting where abiotic features exist, how they vary, and why they matter. Assessment methods, both quantitative and qualitative (Kubalíková et al. 2023; Zwoliński et al. 2023), can be used, for example, to evaluate conservation values (Wolniewicz 2023), relationships with biodiversity (Toivanen et al. 2024), or ecosystem service provision (Santos et al. 2025). Such systematic approach reveals how abiotic variety supports resilience through diversity and redundancy (P1).

In natural systems, this variety provides multiple overlapping pathways for maintaining function under changing conditions (Tukiainen et al. 2025). Varied terrain creates climate refugia, providing spatial insurance against environmental change (Anderson et al. 2023). Different substrates enable various pathways for accessing water and nutrients, offering functional insurance (Stavi et al. 2019). Features that change at different rates provide temporal insurance, maintaining stability when rapid changes destabilize other components (Brazier et al. 2012). The same logic extends to broader human–environment interactions. Geologically varied landscapes support diverse livelihood strategies, providing communities with options to draw on as conditions change. Geopark initiatives such as GEOfood, which leverage geological identity as an economic diversification strategy, illustrate this potential in practice (Çeşmeci et al. 2024). This insurance, however, depends on physical variety being maintained, as human modification of the abiotic environment narrows the options available to both ecosystems and communities, further increasing vulnerability.

Making geodiversity’s physical contributions visible, rather than leaving them implicit in broader ecosystem or landscape concepts, can reveal both the ecological and social dependencies on abiotic variety that remain invisible when physical features are treated as passive backdrop. Two existing frameworks demonstrate what this can look like in practice. First, *Conserving Nature's Stage* (CNS) conservation strategy operates on diversity-as-insurance logic by protecting diverse physical settings that maintain potential habitats for various species (Anderson et al. 2014, 2023). Rather than predicting persistence, this approach protects the stage upon which adaptation occurs. Empirical CNS studies go beyond conceptual claims by explicitly quantifying geodiversity components to test whether physical variety predicts biodiversity patterns (Miller et al. 2024), demonstrating that making geodiversity explicit is both practically feasible and empirically meaningful. Second, the *geosystem services* framework distinguishes benefits delivered directly by abiotic features (construction materials, hydropower, erosion control) from benefits arising through abiotic–biotic interactions (soil formation, habitat variety) (Van Ree et al. 2017; Garcia et al. 2025), making abiotic contributions explicit. By spanning physical to social contributions, from mineral resources to cultural heritage, it complements rather than duplicates traditional ecosystem services approaches. Both two frameworks share a common logic, as they make geodiversity’s contributions nameable and measurable, providing a foundation for informed management decisions.

Yet many physical features are effectively irreversible at human timescales. Once bedrock is quarried, soils stripped, or aquifers depleted, recovery is not possible within timeframes meaningful to societies or ecosystems. Where connections between physical features and the species and processes they support remain undocumented, losses and their ecological consequences are also difficult to detect. Together, these factors make a strong case for protective approaches that map what exists, identify threats, and safeguard adaptation options before they are lost (Hjort et al. 2015; Gordon et al. 2022). Some studies in our review examined geodiversity’s vulnerability to human pressures and climate change (Kubalíková and Balková 2023; Németh et al. 2023; Moradipour et al. 2024), though systematic documentation remains limited.

#### From static stage to dynamic participant

The Conserving Nature’s Stage framework effectively communicates geodiversity’s foundational role through spatial templates for adaptation. However, the stage metaphor can be misleading. Although the framework recognizes that physical stages are dynamic (Gill et al. 2015), the metaphor risks incomplete understanding of how abiotic features actively participate in system transformation (Van Ree and Van Beukering 2016; Beierkuhnlein et al. 2025; Pettorelli and Bridgewater 2025).

Eleven studies in our review found that geodiversity can both facilitate and constrain resilience, depending on context and timescale. The same features may provide resources yet create hazards, or offer protection yet increase exposure ("Nature of the relationship between geodiversity and resilience" section). Understanding these dualities requires distinguishing between analytical perspectives (Table 4): whether geodiversity is viewed as a physical template that supports the resilience of ecological or social systems, or whether geosystems themselves are the subject whose resilience is being assessed (cf. Thoms et al. 2018).

**Table 4:** Analytical perspectives on geodiversity’s role in resilience. The table compares how geodiversity contributes to four distinct conceptualizations of resilience: ecological, engineering, social, and social-ecological resilience

Resilience type	Definition	Geodiversity’s role
Ecological resilience	The magnitude of disturbance a system can absorb before changing its structure by shifting to an alternative stable state, focussing on persistence of system function and relationships rather than return to equilibrium (Holling 1973)	Diverse physical settings provide refugia and multiple pathways for ecosystem reorganization. Geosystems exhibit their own threshold behaviours, with physical changes (e.g. soil erosion) triggering ecosystem shifts to alternative states
Engineering resilience	The speed at which a system returns to a single equilibrium state after disturbance, emphasizing efficiency and predictability of recovery (Holling 1996)	Stable physical foundations can support ecosystem recovery, but many physical disturbances (e.g. erosion, contamination) are effectively irreversible at management timescales, limiting applicability of bounce-back frameworks
Social resilience	The ability of groups or communities to cope with external stresses and disturbances as a result of social, political and environmental change (Adger 2000)	Diverse geological settings provide varied livelihood resources and support cultural values that strengthen social bonds. Changes in geodiversity (e.g. geohazards, resource depletion) create stresses, while access to geological resources affects communities’ adaptive capacity
Social-ecological resilience, or social-environmental resilience	The capacity of a social-ecological system (SES) to absorb disturbance and reorganize while maintaining essential function, structure, identity, and feedbacks through persistence, adaptability (managing along current pathways), and transformability (shifting to new pathways when current ones become untenable) (Walker et al. 2004; Folke et al. 2010)	Geodiversity plays multiple roles in social-environmental systems providing foundations, creating disturbances, mediating ecological and social dynamics, and itself changing over time. Diverse geological settings enable multiple pathways for persistence and adaptation, with physical stability helping maintain essential functions as systems reorganize. Physical changes cascade through coupled components, potentially driving shifts towards fundamentally new system states

These perspectives can engage very different temporal dynamics. When geodiversity functions as a template, components often operate as “slow variables” (Walker et al. 2012). Soil quality and groundwater change gradually relative to ecological dynamics yet control faster processes like crop yields, while topography appears static yet structures settlement patterns over centuries. When analysing geosystem resilience, recovery rates vary from rapid geomorphological processes to effectively non-renewable features. This temporal variability also challenges engineering-based “bounce-back” perspectives (Wied et al. 2020), as many geodiversity components cannot recover at timescales relevant to societies or ecosystems. For geoheritage, this irreversibility is critical, as damaged geosites may permanently lose scientific, educational, or cultural values (Moradipour et al. 2024).

Climate change further disrupts stabilizing temporal relationships. Features that historically have changed over millennia now shift within decades (Brazier et al. 2012; Gordon et al. 2019), an example being permafrost thaw (Hjort et al. 2018). When extreme events occur more frequently, physical systems face shorter recovery periods. Many global tipping points (e.g. changes in cryosphere or ocean circulation) involve geodiversity dimensions whose rates of change are accelerating (Folke et al. 2021). Geodiversity also expands temporal understanding through geological timescales. Geological and palaeoecological archives reveal which configurations have remained stable through past climate shifts and which have crossed critical thresholds (Gray et al. 2013; Gill et al. 2015; Gordon et al. 2019). These archives offer insights that short-term monitoring cannot capture, particularly where biological reference points are lacking (Knudson et al. 2018).

This temporal variability, spanning features that shift within decades to those effectively non-renewable, is what makes managing geodiversity challenging, and what argues for a fundamental shift in management philosophy. Rather than engineering responses that suppress physical variability to maintain single desired states, working “with” physical processes may better support long-term resilience even at the cost of short-term predictability (Prosser et al. 2010; Brazier et al. 2012). Allowing river floodplains to flood or coasts to migrate are examples of this reorientation, reflected in several reviewed studies (Brazier et al. 2012; Harmon and Viles 2013; Matthews 2014; Gordon et al. 2021) and broader landscape resilience principles (Bengtsson et al. 2003). Moreover, mainstreaming geoconservation could support such adaptation, as it explicitly aims to recognize the limits of the physical environment (Crofts et al. 2020).

Current geodiversity assessment approaches focus primarily on spatial patterns (Kubalíková et al. 2023). Moving forward requires frameworks addressing how features change over time, when thresholds are crossed, and how physical variety underpins and shapes human–environment systems across scales, building on geodiversity research’s capacity to recognize abiotic features as both stable foundations for adaptation and dynamic systems that respond to disturbance.

#### From separated domains to integrated systems

Resilience frameworks typically distinguish between natural systems (ecosystems, biodiversity) and social systems (communities, governance, economies), with physical environments subsumed into the ecological domain rather than recognized as bridging both. Geodiversity and geoheritage research challenge this separation by demonstrating that abiotic features are inherently both natural and social. For instance, a volcanic landscape simultaneously operates as a geophysical system, provides fertile soils supporting livelihoods, creates hazards requiring collective response, holds cultural significance, and anchors economic activities, which are interconnected dimensions of the same reality. Treating these as separate domains creates conflicts when mining opposes conservation, tourism development opposes community needs, or hazard management treats geological risks as purely technical problems. Recognizing geodiversity as simultaneously natural and social reframes these conflicts as integration challenges rather than irresolvable trade-offs, and integration becomes possible when the multiple meanings of geological features are built into how they are managed.

The reviewed studies illustrate several ways this works in practice. In geohazard contexts, education makes hazard knowledge accessible to communities rather than leaving it in the hands of technical experts alone, turning features that could be managed around communities into shared resilience resources (De Pascale et al. 2017; Scarlett and Riede 2019; Vereb et al. 2020a; Morino et al. 2022; Arias et al. 2025). The same geological features that create hazards can also become the basis for economic opportunity. The 2014–2015 Fogo eruption, for instance, despite destroying villages, subsequently drove new geotourism development in the affected area (Alfama et al. 2024). Beyond top-down education, communities can take active roles, for instance, in identifying and monitoring sites (Tavares et al. 2015; Fepuleai et al. 2021), or developing geotourism businesses and inventing products centred on local geological identity to enhance economic resilience (Powell et al. 2018; Yuliawati et al. 2024). Furthermore, integrating indigenous geological knowledge with scientific expertise can be used in breaking down the natural/social divide, not just by expanding who participates, but by challenging the assumption that scientific knowledge and community knowledge belong to separate domains in the first place. When communities have lived with and understood geological features for generations, that knowledge should belong in management decisions alongside scientific expertise (Németh and Gravis 2022; Németh and Németh 2023).

As these examples demonstrate, abiotic features connect natural and social systems most effectively when communities are recognized as active participants in their meaning and management rather than passive recipients of technical decisions. As highlighted in the reviewed literature, UNESCO Global Geoparks represent the most developed institutionalization of geodiversity-focussed approach and are examined in the following section as an illustration of what integrated geodiversity–resilience management can look like in practice.

### From concepts to practice: Geodiversity–resilience connections in UNESCO Global Geoparks

**Fig. 4:**
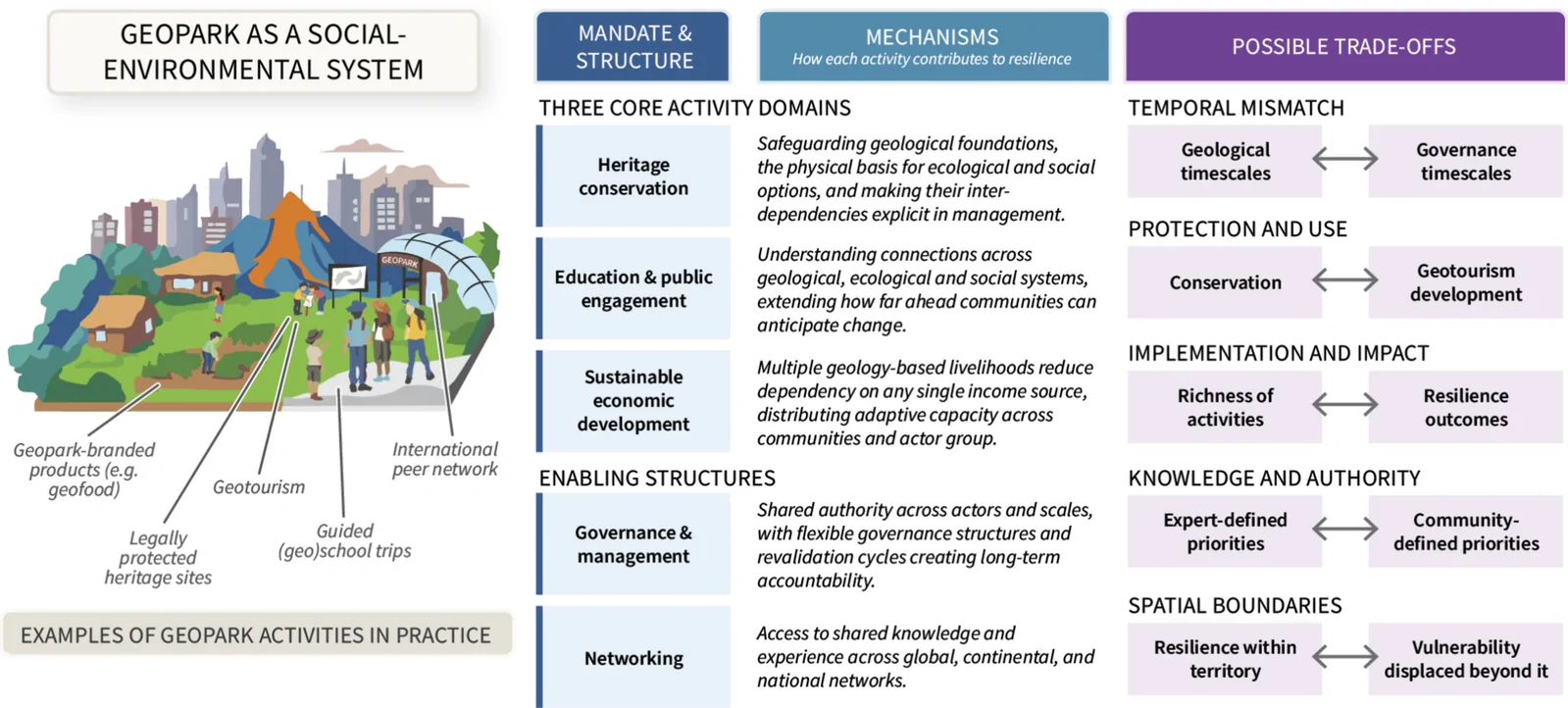
UNESCO Global Geoparks as a context for geodiversity–resilience connections. The left panel frames the geopark as a social-environmental system, and illustrates selected examples of geopark activities. The middle panel maps geoparks’ mandate and structure (three core activity domains and two enabling structures) to their contributions to social-environmental resilience. The right panel identifies examples of potential trade-offs in translating geopark activities into resilience outcomes

UNESCO Global Geoparks have been described as “territories of resilience” (Martini et al. 2022). Their mandate encompasses a holistic concept of protection, education, and sustainable development, pursued through a bottom-up approach that actively involves local communities (UNESCO 2015, 2025). This positions geoparks as an institutional context where geodiversity–resilience connections should be observable in practice. In reality, translating these connections into practice is difficult, as both concepts are highly abstract and most reviewed studies document associations rather than mechanisms. Geoparks nonetheless offer a context where geodiversity is the founding logic of management rather than background (Fig. 4).

The seven principles framework (Biggs et al. 2015) offers a useful lens for unpacking how geopark activities build resilience in practice. The holistic management concept—integrating protection, education, and sustainable development across geological, biological, and cultural heritage—treats the territory as an interconnected system, directly reflecting the principle of fostering complex adaptive systems thinking (P4). Within this system, diversity operates as insurance at multiple levels (P1): diversifying income across geology-linked activities buffers communities when some livelihoods fail, while broad community involvement distributes adaptive capacity across actor groups (P6, Broaden participation). Connectivity (P2) extends beyond the physical to social and knowledge networks, as access to shared experience across international geopark networks means territories facing novel challenges can draw on responses developed elsewhere (cf. COVID-19, Martini et al. 2022). By treating geological heritage loss as effectively irreversible, heritage conservation engages slow variable thinking (P3), with management decisions carrying consequences well beyond the immediate context. Geoeducation builds the capacity to anticipate disturbance and recognize when change in one domain signals risk in another (through hazard literacy, temporal understanding of landscape change, and intergenerational transmission of local knowledge) and represents geoparks’ most direct mechanism for building long-term adaptive capacity (P5, Encourage learning).

Geopark governance is structured in ways that directly reflect polycentric principles (P7, Promote polycentric governance systems), functioning as the enabling architecture within which the other principles operate, shaping whether, for example, learning (P5), stakeholder participation (P6), and slow variable management (P3) translate from design into practice. Authority is distributed across multiple interacting scales (from UNESCO at global level down to individual geoparks, with both national and global peer networks), while the framework deliberately leaves governance structures flexible to suit national and local contexts. At local level, a co-management plan must be developed with local communities, indigenous peoples, and local and regional authorities, with local and indigenous knowledge mandated alongside scientific expertise. The four-year geopark status revalidation cycle adds another dimension, functioning as a governance-level slow variable and feedback mechanism creating accountability across management cycles (P3). Yet the reviewed literature mostly describes these structures rather than examines how they function (Vereb et al. 2020a; Németh et al. 2021; Nemeth et al. 2023), creating an analytical blind spot rather than reflecting an institutional absence. Whether co-management mandates translate into genuine power-sharing, and whether revalidation cycles create meaningful accountability, remains an empirical question to be studied.

Moving from aspirations and associations to practice surfaces tensions that resilience scholars have long flagged (Meerow and Newell 2016). Geodiversity–resilience connections are not politically neutral, and asking resilience of what, for whom, and at what scale matters. The timescales at which geodiversity builds resilience (geological and intergenerational) may not align with institutional accountability cycles that reward short-term measurable outcomes (Knudson et al. 2018), and sectoral governance fragmentation means decisions about what to protect and for whom reflect particular interests rather than the integrated way communities experience their environment. These tensions are well illustrated by geotourism, which functions simultaneously as the economic “engine” of geopark sustainable development and a potential threat to the geological heritage it depends on (cf. Gordon 2023). More broadly, the richness of geopark activities does not automatically translate into actual resilience, and documenting what geoparks do is considerably easier than demonstrating what difference it makes. Resilience built within a bounded geopark territory may also displace rather than reduce vulnerability beyond it.

In geoparks, all seven principles can be identified on paper, yet whether they actually function as resilience-building mechanisms remains unexamined. This is a gap that reflects the broader challenge of moving from geodiversity–resilience associations to demonstrated mechanisms. As one of the few contexts where geodiversity-centred governance actually exists, geoparks offer a uniquely valuable setting for that work.

### Barriers and pathways forward

Our analysis reveals that geodiversity research already engages extensively with resilience principles, yet these connections remain largely invisible to the broader resilience community. Three mutually reinforcing barriers prevent integration: visibility, evidence, and institutional gaps.

#### Bridging the visibility gap: From fragmentation to shared language

Both geodiversity and social-ecological resilience are relatively young fields, emerging within the last few decades, making their limited overlap understandable. Yet both now address the same sustainability challenges, creating opportunities for productive dialogue.

The invisibility stems from problems on both sides. Earth science subfields (geology, pedology, geomorphology, hydrology) remain fragmented, each using distinct terminology. Meanwhile, resilience literature lumps physical components under vague umbrella terms. When we searched 1011 social-ecological resilience documents, geology appeared in only 3, geomorphology in 5, hydrology in 14, and soil in 41, whereas climate dominated with 327 mentions.^1^ Specific physical features hide beneath broad terms like “environmental”, “ecosystems”, or “landscapes”. Similar phenomena manifests in our review as well: only 17 of 90 geodiversity studies explicitly engaged with resilience theory, yet 46 demonstrated implicit understanding. Our search strategy itself reflects this fragmentation. Focussing on geodiversity and geoheritage as marker keywords necessarily excludes Earth science research addressing the same physical features under different terminology. A complementary review examining how geomorphology, soils, and hydrology feature in social-ecological resilience research would be a valuable next step.

Bridging this gap requires effort from both fields. Understanding the disconnect requires examining how resilience literature addresses the physical environment and how resilience is conceptualized across Earth science disciplines (e.g. Corstanje et al. 2015; Thoms et al. 2018), to truly understand whether geodiversity is overlooked or simply described differently. Geodiversity researchers could strengthen connections by explicitly framing work using resilience concepts and clarifying conceptual distinctions, as our review found terms like resilience, sustainability, and vulnerability often used interchangeably (cf. Marchese et al. 2018). Resilience research would benefit from granting physical diversity the systematic attention often given to biodiversity. Fields like socio-hydrology (Di Baldassarre et al. 2019) and social soil sciences (Coelho Junior et al. 2023) demonstrate that productive integration is already happening. The seven principles framework offers common analytical language that makes geodiversity’s contributions recognizable across disciplinary boundaries. And as "From concepts to practice: Geodiversity–resilience connections in UNESCO Global Geoparks" section shows, institutional contexts like UNESCO Global Geoparks can provide the practical setting where such integration moves from concept to management reality.

#### Strengthening the evidence base: From documentation to mechanism

Geodiversity research effectively documents what the physical environment provides but less often examines the mechanisms through which these contributions enhance resilience—that is, how, under what conditions, and at what scales they translate into resilience outcomes. Our results illustrate this gap, as 77 studies portrayed geodiversity as facilitating resilience, while only 11 examined mixed or context-dependent relationships (out of 90 studies).

This pattern primarily reflects a conceptual premise within geodiversity research where demonstrating the benefits and services of geodiversity itself might be a research goal. Studies therefore tend to select contexts where positive relationships are expected, and resilience arguments remain linked to broad physical heterogeneity rather than to specific features, processes, or conditions. Negative effects are consequently more visible in adjacent fields such as natural hazard research, where abiotic processes are not filtered through a services or heritage lens.

While the premise that “more geodiversity equals more resilience” has sound theoretical grounding—diversity and redundancy being a core principle of resilience thinking (Biggs et al. 2015)—strengthening the evidence base of actual resilience outcomes requires moving beyond it. Not abandoning it, but complementing it with investigation of how context, spatial and temporal scale, disturbance type, and competing uses shape whether and how geodiversity contributions operate (see e.g. Gill et al. 2015 on scale- and context-dependence within the CNS framework). This means developing evidence that is actionable in specific governance and management settings rather than demonstrative of geodiversity’s value in general.

The challenge intensifies because the same features can both support and undermine resilience depending on context and scale. Context-dependence is well illustrated, for instance, by how geological hazards can simultaneously threaten communities and, over longer timescales, form the basis of geoheritage that strengthens community resilience (Németh 2025). Considering scale, the same geological formation may appear uniformly beneficial at a coarse spatial scale while being simultaneously contested at finer scales: valued as a strategic mineral resource at supranational level, managed as a conservation asset at national level, and experienced as a foundation for community identity and livelihoods locally. Negotiating such trade-offs requires governance mechanisms that can operate across scales and reconcile competing values, and currently little institutional infrastructure exists to do this specifically for geodiversity.

Several practical contexts illustrate where stronger evidence would make a difference. In land-use planning, geodiversity is typically treated as something to be managed around (e.g. slope stability, flood risk, mineral extraction) rather than assessed and valued as diversity in its own right, in contrast to how biodiversity is treated. In climate adaptation, heterogeneous topography, soils, and hydrology provide physical refugia and buffering capacity that adaptation frameworks tend to treat as background conditions rather than resources to be actively maintained (cf. the idea of Conserving Nature’s Stage). In disaster risk reduction, studies of volcanic geoheritage show how treating geological features as heritage rather than pure threats transforms communities’ relationships to hazard landscapes, building preparedness through understanding rather than avoidance (Németh et al. 2023; Arias et al. 2025).

A productive way forward is to treat geodiversity as an umbrella term analogous to biodiversity, where different physical components become relevant for different questions and contexts. Strengthening the evidence base therefore requires developing protocols for assessing geodiversity contributions to specific resilience outcomes, and systematic research on conditions under which physical variety enhances versus undermines resilience. Frameworks such as Conserving Nature’s Stage and geosystem services provide ready analytical foundations for this work.

#### Building institutional infrastructure: From potential to practice

The governance gap presents the most pressing challenge for operationalizing geodiversity–resilience connections. Managing the physical environment is inherently political, involving competing values and interests, and without geodiversity-specific institutional infrastructure, the physical variety of nature risks remaining invisible in environmental governance and decision-making across the temporal and spatial scales that physical systems require.

Biodiversity benefits from extensive institutional infrastructure (international conventions, monitoring frameworks, assessment reports, and funding mechanisms) that makes it visible in decision-making. Geodiversity lacks comparable architecture, instead fragmenting across resource-specific governance for water, minerals, or soils. While individual geosites can be protected through national legislation or the World Heritage Convention, no widely implemented monitoring or assessment frameworks exist for geodiversity comparable to those developed for biodiversity (Crofts et al. 2020; Prosser et al. 2025). Without such frameworks, interacting pressures on geodiversity remain invisible until thresholds are crossed.

Recent developments suggest broader change is possible. Essential Geodiversity Variables (Schrodt et al. 2019, 2024) provide systematic monitoring frameworks compatible with Essential Biodiversity Variables. The IUCN Guidelines for Geoconservation (Crofts et al. 2020) offer best-practice guidance for managing geodiversity within protected areas, explicitly linking geological and biological conservation. Additionally, the Key Geoheritage Areas initiative (Brilha et al. 2025; parallel to Key Biodiversity Areas) and the establishment of International Geodiversity Day (Zwoliński et al. 2023; Hooton et al. 2025) demonstrate that international frameworks and recognitions for geodiversity are increasingly taking shape. UNESCO Global Geoparks complement these frameworks by demonstrating what geodiversity-centred governance already looks like in practice, making them the most operationally developed component of an institutional infrastructure for geodiversity that is otherwise still taking shape.

## Conclusions

When nature is equated primarily with biodiversity, the physical foundations upon which ecosystems and societies depend risk being overlooked. Our systematic scoping review demonstrates that geodiversity research functions as a hidden knowledge base for resilience thinking—one that engages substantially with core resilience principles yet remains largely invisible in broader sustainability discourse.

Geodiversity enriches resilience thinking in three ways: making Earth’s abiotic foundations explicit, recognizing physical features as active participants in system change, and showing these features are simultaneously natural phenomena and social resources. However, three barriers prevent this potential from translating into practice: disciplinary fragmentation obscures connections, a bias towards description over explanation weakens the evidence base, and institutional gaps limit systematic inclusion in governance.

Yet pathways forward exist. Emerging initiatives (Essential Geodiversity Variables, Key Geoheritage Areas, UNESCO Global Geoparks) are building the institutional infrastructure geodiversity has lacked. Frameworks like Conserving Nature’s Stage and geosystem services offer ready tools for incorporating physical dimensions into resilience planning, and mainstreaming geoconservation within nature conservation policy would extend these into established governance frameworks.

As sustainability challenges demand integrated perspectives, making explicit what has remained implicit opens pathways towards holistic understanding of how physical, ecological, and social dimensions intertwine. Realizing this potential requires building cross-disciplinary visibility, strengthening mechanistic evidence, and developing governance structures that treat geodiversity as an active component of social-ecological resilience.

## Supplementary Information

Below is the link to the electronic supplementary material.

Supplementary file1 (PDF 491 KB)

## Footnotes

1 Scopus search on “social-ecological resilience” OR “socio-ecological resilience” AND geolog*/soil*/geomorpholog*/hydolog*/climat* (in abstract, titles and keywords) on 28th Nov 2025. Search on “social-ecological resilience” OR “socio-ecological resilience” alone yielded 1011 results.
