# Supplementary material for: Geodiversity and resilience: A scoping review
Source: Ambio. 2026 May 3;55(12):2815–35. doi: 10.1007/s13280-026-02409-8 (PMC13731613; doi:10.1007/s13280-026-02409-8)
Supplement: Supplementary file 1 [file 13280_2026_2409_MOESM1_ESM.pdf]

Supplementary files

Title: Geodiversity and resilience: a scoping review

Journal: Ambio

Authors: Toivanen, Maija*; Santos, Daniel; Räsänen, Aleksi

*Corresponding author. Affiliation: Geography Research Unit, University of Oulu, Finland. E-mail: maija.toivanen@oulu.fi

Contents of this file

Table S1. Data analysis template for systematic review. (Page 2)

Table S2. PRISMA-ScR checklist (Page 3)

Table S3. Summary of reviewed literature and results. (Page 5)

Table S4. Summary of resilience definitions and theoretical frameworks in studies with explicit engagement. (Page 12)

References

Table S1. Data analysis template for systematic review used in Covidence software.

Category	Item	Description	Response options or format
**General publication characteristics**	Study title		Free text
	Authors		Free text
	Year of publication		Year
	Journal title		Free text
	Type of paper	E.g. empirical, theoretical/conceptual, methodological, review, argumentative/position paper, editorial/commentary, other	Free text
	Study summary	Brief summary of the paper's main purpose, research question, or central argument (2-3 sentences)	Free text
	Study area/region	Geographic location or region of focus	Free text
	Geopark connection	Is the study conducted in or related to a geopark?	Yes / No
**Geodiversity focus**	Main geodiversity focus(es)	Primary geodiversity approaches or perspectives that characterize the paper's main focus	Geodiversity / Geoheritage / Geoconservation / Geotourism / Geosystem services / Other
**Resilience conceptualization**	How is resilience addressed?*	Level of engagement with resilience concepts	Explicit engagement / Implicit understanding / Mentioned only
	Resilience description**	How resilience is mentioned or discussed (where it appears, how it's used). For explicit definitions, include direct quotes with references.	Free text
	Implicit connections	Aspects relating to resilience concepts without explicit resilience terminology	Free text
**Geodiversity–resilience relationship**	Relationship description	How the authors connect geodiversity and resilience (2-3 sentences)	Free text
	Strength of evidence	Quality and clarity of evidence for the relationship	Strong / Moderate / Weak / No explicit connection made
	Evidence quality and key references	Type of evidence (empirical, theoretical, speculative) and main supporting citations	Free text
	Study limitations	Limitations affecting the strength or scope of geodiversity-resilience findings	Free text
	Geodiversity role*	How geodiversity is framed in relation to resilience	Facilitator / Constraint / Mixed-ambiguous / Other
	Role explanation	Brief explanation with examples from the paper	Free text
	Spatial scale	Scale at which geodiversity-resilience connection is examined	Site or local / Regional / National / Global / Multiple scales / Unclear
**Alignment with resilience principles (P1–P7)**	Principles addressed*	Connections to the seven principles for building resilience in social-ecological systems (Biggs et al. 2012, 2015)	P1 Maintain diversity and redundancy / P2 Manage connectivity / P3 Manage slow variables and feedbacks / P4 Foster complex adaptive systems thinking / P5 Encourage learning / P6 Broaden participation / P7 Promote polycentric governance systems / None applicable / Other
	Explanation for each principle	How each selected principle is addressed, with specific examples	Free text

* The results are summarized in Table S2.

** Descriptions of explicit engagement are summarized in Table S3.

Table S2. PRISMA-ScR checklist following Tricco et al. (2018).

ITEM	SECTION	REPORTED
**TITLE**		
1	Title	Title, abstract and keywords and methods section identify it as a systematic scoping review.
**ABSTRACT**		
2	Structured summary	The abstract follows journal formatting guidelines rather than a structured PRISMA format. However, it covers background, objectives, sources of evidence, results, and conclusions Eligibility criteria and charting methods are not explicitly stated in the abstract but are detailed in the methods section.
**INTRODUCTION**		
3	Rationale	The rationale of research is described in the introduction. The scoping review approach is justified in section 2.1 as appropriate for capturing the breadth of this emerging and heterogeneous research area where terminology is inconsistent across disciplines.
4	Objectives	Two research questions (RQ1 and RQ2) are explicitly stated at the end of the introduction.
**METHODS**		
5	Protocol and registration	No review protocol was registered, which we acknowledge as a limitation of our methodological transparency. The data extraction template used in the review is provided in full as Table S1.
6	Eligibility criteria	Eligibility criteria are described in section 2.1. Studies were included if they addressed geodiversity and made explicit or implicit connections to resilience, across all study designs, publication types, and geographic contexts. Studies were excluded if they lacked substantive focus on either geodiversity or resilience, mentioned both concepts without connecting them, or were inaccessible. The broad inclusion criteria reflect the scoping review aim of capturing the breadth of an emerging research area. All included studies were in English with no date restrictions.
7	Information sources*	Scopus and Web of Science Core Collection were searched on 24th March 2025. Backward citation screening of studies forwarded to full-text screening was also conducted to address geodiversity terminology inconsistencies. Author contact via Research Gate website was attempted for inaccessible studies.
8	Search	Full search strings are provided in section 2.1. First string: (“geodiversity” AND “resilience”); second string: (“geoheritage” AND “resilience”). “Geodiversity” and “geoheritage” were limited to title, abstract, and keywords, and “resilience" was searched across all fields. Searches were conducted in both Scopus and Web of Science Core Collection on 24th March 2025.
9	Selection of sources of evidence†	Described in section 2.1. Screening and eligibility assessment were conducted using Covidence software. Studies were first screened by title and abstract, then assessed at full-text stage against inclusion and exclusion criteria. A PRISMA flow diagram is provided as Figure 2.
10	Data charting process‡	Described in section 2.2. A structured data extraction template was developed and piloted on approximately 40% of studies before refinement. Data were extracted by one reviewer, with the template and analytical approach continuously developed and refined through discussion among the author group. Author contact via ResearchGate was attempted for inaccessible studies.
11	Data items	All variables are listed and defined in the data extraction template provided as Table S1, covering publication characteristics, geodiversity focus, resilience conceptualization, geodiversity–resilience relationships, and alignment with the seven resilience principles.
12	Critical appraisal of individual sources of evidence§	Evidence strength was assessed for each geodiversity-resilience relationship during data extraction (strong/moderate/weak/no explicit connection, Table S1) but was not used as an exclusion criterion and did not reveal patterns sufficiently distinct to warrant separate reporting (consistent with scoping review methodology, which maps breadth of evidence rather than appraising its quality).
13	Synthesis of results	Described in section 2.2. Data were first analysed descriptively to characterize publication trends, geographic distribution, study designs, and disciplinary contexts. Analysis was then structured around the two research questions: RQ1 examined theoretical engagement with resilience concepts across its full disciplinary range, and RQ2 examined geodiversity–resilience relationships and their alignment with the seven principles framework.

RESULTS		
14	Selection of sources of evidence	Numbers of sources screened, assessed, and included are reported in section 3.1 and presented visually in the PRISMA flow diagram (Figure 2). Full-text screening resulted in 90 studies meeting inclusion criteria.
15	Characteristics of sources of evidence	Characteristics of all 90 included studies are summarized in Table S3, including publication details, resilience engagement level, geodiversity role, and coded resilience principles. Full citations are provided in the reference list.
16	Critical appraisal within sources of evidence	Evidence strength was assessed during data extraction, but patterns did not warrant separate reporting. See item 12.
17	Results of individual sources of evidence	Charted data for each included study are presented in Table S3. Results relevant to RQ1 are reported in section 3.2 and Table 2, with detailed resilience definitions in Table S4. Results relevant to RQ2 are reported in sections 3.3 and 3.4.
18	Synthesis of results	Results are summarised in sections 3.2–3.4 and synthesized in the discussion (section 4), structured around the two research questions. Key findings are also summarised in the abstract and conclusions.
**DISCUSSION**		
19	Summary of evidence	Main results are summarised at the opening of section 4, linking findings back to both research questions.
20	Limitations	Methodological limitations are discussed in section 2.4, including: search strategy focused on geodiversity and geoheritage terminology (excluding abiotic diversity research using terms like geomorphology, soils, or hydrology), single-reviewer data extraction mitigated through iterative template refinement and re-analysis, restriction to two databases compensated by backward citation screening, English-language restriction, and lack of prospective protocol registration.
21	Conclusions	General interpretation is provided in section 4 and section 5 (conclusions). Implications for resilience thinking are discussed in section 4.1 (three perspectives on geodiversity's contributions), governance and institutional practice in section 4.2 (UNESCO Global Geoparks as proof of concept), and barriers and pathways forward in section 4.3. Next steps identified include building visibility across disciplines, shifting the evidence base from documentation toward mechanisms, and developing institutional infrastructure for geodiversity governance.
**FUNDING**		
22	Funding	Funding for the scoping review is acknowledged in the manuscript funding statement. Funding sources for individual included studies were not systematically extracted as part of the review.

JBI = Joanna Briggs Institute; PRISMA-ScR = Preferred Reporting Items for Systematic reviews and Meta-Analyses extension for Scoping Reviews.

* Where *sources of evidence* (see second footnote) are compiled from, such as bibliographic databases, social media platforms, and Web sites.

† A more inclusive/heterogeneous term used to account for the different types of evidence or data sources (e.g., quantitative and/or qualitative research, expert opinion, and policy documents) that may be eligible in a scoping review as opposed to only studies. This is not to be confused with *information sources* (see first footnote).

‡ The frameworks by Arksey and O'Malley (6) and Levac and colleagues (7) and the JBI guidance (4, 5) refer to the process of data extraction in a scoping review as data charting.

§ The process of systematically examining research evidence to assess its validity, results, and relevance before using it to inform a decision. This term is used for items 12 and 19 instead of "risk of bias" (which is more applicable to systematic reviews of interventions) to include and acknowledge the various sources of evidence that may be used in a scoping review (e.g., quantitative and/or qualitative research, expert opinion, and policy document).

Table S3. List of reviewed literature and summary of main results, including resilience engagement, nature of geodiversity–resilience relationship, and coded resilience principles (P1–P7) in each study.

Authors, year, journal title	Study title	Resilience engagement	Geodiversity's role	P1	P2	P3	P4	P5	P6	P7
Agastya et al. 2024, *International Journal of Geoheritage and Parks*	Model media learning for disaster risk reduction in the Batur UNESCO Global Geopark	Mentioned only	Facilitator	1	0	0	0	1	1	0
Alahuhta et al. 2019, *Nature Ecology & Evolution*	Geodiversity–biodiversity relationship needs more empirical evidence	Mentioned only	Facilitator	1	0	0	0	0	0	0
Alberico et al. 2023, *Geoheritage*	The Effectiveness of Geotrails to Support Sustainable Development in the Campi Flegrei Active Volcanic Area	Explicit engagement	Facilitator	1	0	0	0	1	1	0
Aleksova et al. 2024a, *Geoheritage*	Palaeovolcanic Geoheritage from Volcano Geology Perspective within Earth's Geosystems: Geoeducation of the Potential Geopark Kratovo-Zletovo (North Macedonia)	Mentioned only	Facilitator	0	0	0	0	1	0	0
Aleksova et al. 2024b, *Geosciences*	Preliminary Assessment of Geohazards' Impacts on Geodiversity in the Kratovska Reka Catchment (North Macedonia)	Mentioned only	Mixed	1	0	0	0	0	0	0
Alfama et al. 2024, *Geoheritage*	The Challenging Nature of Volcanic Heritage: The Fogo Island (Cabo Verde, W Africa)	Implicit understanding	Mixed	1	0	0	0	1	1	0
Alyaqout and Anzah 2025, *International Journal of Geoheritage and Parks*	Application of bivariate mapping to assess geodiversity and its geomorphic constraints: A case study in Kuwait	Mentioned only	Facilitator	1	0	0	0	0	0	0
Anderson et al. 2014, *Conservation Biology*	Estimating Climate Resilience for Conservation across Geophysical Settings	Explicit engagement	Facilitator	1	1	0	0	0	0	0
Anderson et al. 2015, *Conservation Biology*	Case studies of conservation plans that incorporate geodiversity	Implicit understanding	Facilitator	1	1	1	0	0	0	0
Anderson et al. 2023, *Proceedings of the National Academy of Sciences*	A resilient and connected network of sites to sustain biodiversity under a changing climate	Explicit engagement	Facilitator	1	1	1	0	0	0	0
Arias et al. 2025, *International Journal of Geoheritage and Parks*	Volcanic geoheritage in Arequipa, Southern Peru: Assessment of pilot geosites for geohazard resilience	Implicit understanding	Facilitator	1	0	0	0	1	1	0
Avagyan et al. 2023, *Geoheritage*	The Potential for a Geohazard-Related Geopark in Armenia	Mentioned only	Unclear	0	0	0	0	1	1	0
Baitalik 2024, *Geoheritage*	Why Geoheritage Matters in Geoconservation Strategies: A Case Study from the Khowai Badlands in West Bengal, India	Explicit engagement	Facilitator	1	0	0	1	0	1	0
Basson et al. 2023, *Frontiers in Environmental Science*	Subsurface geodiversity determines shrub resilience vs. mortality under long-term droughts in the Israeli Negev drylands	Implicit understanding	Facilitator	1	0	1	0	0	0	0

Benito et al. 2020, *Frontiers in Ecology and Evolution*	Spatial and Temporal Ecological Uniqueness of Andean Diatom Communities Are Correlated With Climate, Geodiversity and Long-Term Limnological Change	Mentioned only	Mixed	0	0	0	0	0	0	0
Brazier et al. 2012, *Scottish Geographical Journal*	Making Space for Nature in a Changing Climate: The Role of Geodiversity in Biodiversity Conservation	Implicit understanding	Facilitator	1	1	1	1	0	0	0
Brown et al. 2012, *Scottish Geographical Journal*	Geodiversity, Conservation and Climate Change: Key Principles for Adaptation	Mentioned only	Facilitator	1	1	1	0	1	1	0
Comer et al. 2015, *Conservation Biology*	Incorporating geodiversity into conservation decisions	Implicit understanding	Facilitator	1	1	1	0	0	0	0
Crofts 2019, *International Journal of Geoheritage and Parks*	Linking geoconservation with biodiversity conservation in protected areas	Implicit understanding	Facilitator	1	1	0	0	0	0	0
Dakhil et al. 2021, *Environmental Research Letters*	Richness patterns of endemic and threatened conifers in south-west China: topographic-soil fertility explanation	Mentioned only	Facilitator	1	0	0	0	0	0	0
Das et al. 2024, *Geoheritage*	Geoheritage of Charaideo Moidams (Assam, India): a Tale of Human Resilience and Man-Environment Relationship	Implicit understanding	Facilitator	1	0	1	0	1	0	0
De Falco et al. 2021, *Scientific Reports*	Geodiversity impacts plant community structure in a semi-arid region	Implicit understanding	Facilitator	1	1	1	0	0	0	0
De Pascale et al. 2017, *Natural Hazards*	Resilience and seismic risk perception at school: a geoethical experiment in Aiello Calabro, southern Italy	Explicit engagement	Mixed	1	0	0	1	1	1	0
Dos Santos Pereira et al. 2022, *Catena*	Detrimental effects of tourist trails on soil system dynamics in Ubatuba Municipality, São Paulo State, Brazil	Mentioned only	Facilitator	0	0	0	0	0	0	0
Everard and Quinn 2015, *International Journal of River Basin Management*	Realising the value of fluvial geomorphology	Implicit understanding	Facilitator	1	1	1	0	0	1	0
Fassoulas et al. 2022, *Geosciences*	Digital Tools to Serve Geotourism and Sustainable Development at Psiloritis UNESCO Global Geopark in COVID Times and Beyond	Mentioned only	Facilitator	1	0	0	0	1	1	0
Fepuleai et al. 2021, *Geoheritage*	Geopark Impact for the Resilience of Communities in Samoa, SW Pacific	Implicit understanding	Facilitator	1	0	0	0	1	1	0
Ferrer et al. 2021, *Resources Policy*	The anthropocene landscape and ecosystem services in the closure of sand mining: Paraíba do Sul River basin – Brazil	Implicit understanding	Facilitator	1	0	1	0	1	0	0
Giardino et al. 2022, *Heritage*	ERASMUS+ Strategic Partnerships between UNESCO Global Geoparks, Schools, and Research Institutions: A Window of Opportunity for Geoheritage Enhancement and Geoscience Education	Mentioned only	Facilitator	1	0	0	0	1	1	0

Gill et al. 2015, *Conservation Biology*	A 2.5-million-year perspective on coarse-filter strategies for conserving nature's stage	Implicit understanding	Facilitator	1	1	1	1	0	0	0
Gonçalves et al. 2024, *International Journal of Geoheritage and Parks*	Assessment of forest fires impacts on geoheritage: A study in the Estrela UNESCO Global Geopark, Portugal	Implicit understanding	Facilitator	1	0	1	0	0	0	0
Gordon et al. 2012, *Proceedings of the Geologists' Association*	Engaging with geodiversity—why it matters	Implicit understanding	Facilitator	1	1	1	0	1	0	0
Gordon and Barron 2013, *Scottish Journal of Geology*	The role of geodiversity in delivering ecosystem services and benefits in Scotland	Implicit understanding	Facilitator	1	0	1	0	1	0	0
Gordon et al. 2018, *Geoheritage*	Enhancing the Role of Geoconservation in Protected Area Management and Nature Conservation	Implicit understanding	Facilitator	1	0	1	1	1	0	0
Gordon 2019, *International Journal of Geoheritage and Parks*	Geoconservation principles and protected area management	Implicit understanding	Facilitator	1	1	1	1	1	0	0
Gordon et al. 2019, *Earth and Environmental Science Transactions of the Royal Society of Edinburgh*	Advances in Quaternary studies and geomorphology in Scotland: implications for geoconservation	Mentioned only	Facilitator	1	0	0	0	0	0	0
Gordon et al. 2021, *International Journal of Geoheritage and Parks*	Including geoconservation in the management of protected and conserved areas matters for all of nature and people	Implicit understanding	Facilitator	1	1	0	0	0	1	0
Gordon et al. 2022, *Geoheritage*	Planning for Climate Change Impacts on Geoheritage Interests in Protected and Conserved Areas	Explicit engagement	Facilitator	1	1	0	1	1	0	0
Gordon et al. 2023, *Proceedings of the Geologists' Association*	Valuing the Quaternary – Nature conservation and geoheritage	Implicit understanding	Facilitator	1	0	1	0	1	0	0
Gordon 2023, *Tourism and Hospitality*	Climate Change and Geotourism: Impacts, Challenges, and Opportunities	Implicit understanding	Facilitator	1	1	1	0	1	1	0
Gray et al. 2013, *Proceedings of the Geologists' Association*	Geodiversity and the ecosystem approach: the contribution of geoscience in delivering integrated environmental management	Implicit understanding	Facilitator	1	1	1	0	1	0	0
Gray 2024, *Philophical Transactions of the Royal Society A*	Case studies associated with the 10 major geodiversity-related topics	Mentioned only	Facilitator	1	0	0	0	1	1	0
Guilbaud et al. 2021, *Geoheritage*	Xitle Volcano Geoheritage, Mexico City: Raising Awareness of Natural Hazards and Environmental Sustainability in Active Volcanic Areas	Implicit understanding	Facilitator	1	0	1	1	1	0	0
Guilbaud et al. 2024, *Geofísica Internacional*	The multiple values of urban geosites: El Arenal and Viaje a la Naturaleza parks in Mexico City	Mentioned only	Facilitator	0	0	0	0	1	0	0
Gupta and Dixit 2024, *Environmental Monitoring and Assessment*	Quantification of geomorphodiversity and its spatial distribution with the flood inundation areas for Assam, India	Mentioned only	Mixed	0	0	0	0	0	0	0

Hamidova et al. 2024, *Land*	Nature-Based Solution Integration to Enhance Urban Geomorphological Mapping: A Methodological Approach	Mentioned only	Facilitator	0	0	0	1	1	0	0
Harmon and Viles 2013, *Environmental Systems and Decisions*	Beyond geomorphosites: trade-offs, optimization, and networking in heritage landscapes	Implicit understanding	Unclear	1	1	0	0	1	1	0
Hoyland et al. 2024, *International Journal of Wildland Fire*	Fire and geodiversity	Mentioned only	Facilitator	1	0	1	1	1	0	0
Knudson et al. 2018, *Nature Climate Change*	Appraising geodiversity and cultural diversity approaches to building resilience through conservation	Explicit engagement	Facilitator	1	1	1	1	0	0	0
Koupatsiaris and Drinia 2024, *Sustainability*	Expanding Geoethics: Interrelations with Geoenvironmental Education and Sense of Place	Implicit understanding	Facilitator	0	0	0	0	1	1	0
Kubalíková et al. 2023, *Geological Society, London, Special Publications*	Visages of geodiversity and geoheritage: a multidisciplinary approach to valuing, conserving and managing abiotic nature	Implicit understanding	Facilitator	0	0	0	0	1	1	0
Kubalíková and Balková 2023, *Environmental Impact Assessment Review*	Two-level assessment of threats to geodiversity and geoheritage: A case study from Hády quarries (Brno, Czech Republic)	Mentioned only	Facilitator	0	0	1	0	1	1	0
Kärnä et al. 2019, *Landscape Ecology*	Does catchment geodiversity foster stream biodiversity?	Mentioned only	Facilitator	1	0	0	0	0	0	0
Magness et al. 2018, *Landscape Ecology*	Using topographic geodiversity to connect conservation lands in the Central Yukon, Alaska	Implicit understanding	Facilitator	1	1	0	0	0	0	0
Manríquez et al. 2019, *eco.mont*	Geodiversity meets Biodiversity: a landscape approach for biogeocultural conservation and governance in Mediterranean central Chile	Mentioned only	Facilitator	1	0	0	0	0	1	1
Mariani and Melis 2022, *Geoheritage*	The Potential for Valorisation of Archaeo-geosites Through Climate Change: Exploratory Study of the Nora Site (Sardinia, Italy)	Mentioned only	Mixed	1	0	1	0	1	1	0
Matthews 2014, *Geoheritage*	Integrating Geoconservation and Biodiversity Conservation: Theoretical Foundations and Conservation Recommendations in a European Union Context	Implicit understanding	Facilitator	1	0	1	1	0	0	0
Mehdipour Ghazi and Audra 2022, *Geoheritage*	Travertine Park in Azarshahr (NW Iran): an Opportunity for Geoheritage Conservation and Diminishing Geohazards Risk	Implicit understanding	Mixed	0	0	1	0	1	1	0
Miller et al. 2024, *Philosophical Transactions of the Royal Society A*	A test of Conserving Nature's Stage: protecting a diversity of geophysical traits can also support a diversity of species at a landscape scale	Mentioned only	Facilitator	1	0	0	1	0	0	0
Moradipour et al. 2024, *Geoheritage*	Geoheritage Resilience Assessment for Geoconservation Planning in Lorestan Province, Iran	Explicit engagement	Facilitator	0	0	1	1	1	1	0

Morante-Carballo et al. 2023, *Geography and Sustainability*	Evaluation of the potential of coastal cliffs as geosites for the promotion of geotourism	Mentioned only	Facilitator	1	1	0	0	1	1	0
Moretti et al. 2021, *Geosciences*	The Basse-Terre Island of Guadeloupe (Eastern Caribbean, France) and Its Volcanic-Hydrothermal Geodiversity: A Case Study of Challenges, Perspectives, and New Paradigms for Resilience and Sustainability on Volcanic Islands	Explicit engagement	Mixed	1	0	1	1	1	0	0
Morino et al. 2022, *Frontiers in Earth Science*	Landslides, a Key Landform in the Global Geological Heritage	Implicit understanding	Mixed	0	0	0	0	1	0	0
Németh et al. 2021a, *Geoheritage*	Geoheritage Conservation: Systematic Mapping Study for Conceptual Synthesis	Implicit understanding	Facilitator	0	0	0	0	0	1	1
Németh et al. 2021b, *Sustainability*	Dilemma of Geoconservation of Monogenetic Volcanic Sites under Fast Urbanization and Infrastructure Developments with Special Relevance to the Auckland Volcanic Field, New Zealand	Implicit understanding	Facilitator	0	0	0	0	1	1	0
Németh and Gravis 2022, *International Journal of Geoheritage and Parks*	Geoheritage and geodiversity elements of the SW Pacific: A conceptual framework	Implicit understanding	Facilitator	1	0	1	1	1	1	0
Nemeth et al. 2023, *Geoconservation Research*	The Vanishing Volcanic Geoheritage of a Key Scoria Cone and its Significance in Volcanic Hazard Resilience of the Active Monogenetic Volcanic Field near Al Madinah, Kingdom of Saudi Arabia	Implicit understanding	Facilitator	0	0	0	0	0	0	0
Németh and Németh 2023, *Geological Society, London, Special Publications*	Spatial decision-making support for geoheritage conservation in the urban and indigenous environment of the Auckland Volcanic Field, New Zealand	Implicit understanding	Facilitator	1	0	0	0	1	1	0
Pelacani et al. 2024, *Ecological Indicators*	Combining biodiversity and geodiversity on landscape scale: A novel approach using rare earth elements and spatial distribution models in an agricultural Mediterranean landscape	Implicit understanding	Facilitator	1	1	1	0	0	0	0
Petrosino et al. 2019, *Geoheritage*	Enhancing Social Resilience Through Fruition of Geological Heritage in the Vesuvio National Park	Explicit engagement	Facilitator	0	0	0	0	1	1	0
Pijet-Migoń and Migoń 2024, *Sustainability*	New Geo- and Mining Heritage-Based Tourist Destinations in the Sudetes (SW Poland)—Towards More Effective Resilience of Local Communities	Explicit engagement	Facilitator	1	1	0	0	1	1	1
Powell et al. 2018, *Tourism Planning & Development*	Examining Community Resilience to Assist in Sustainable Tourism Development Planning in Dong Van Karst Plateau Geopark, Vietnam	Explicit engagement	Facilitator	1	0	1	0	1	1	0

Rentier et al. 2024, *Renewable and Sustainable Energy Reviews*	Lithium brine mining affects geodiversity and Sustainable Development Goals	Implicit understanding	Facilitator	1	1	1	0	0	0	0
Roque Guerrero et al. 2020, *Sustainability*	Mapping Potential Zones for Ecotourism Ecosystem Services as a Tool to Promote Landscape Resilience and Development in a Brazilian Municipality	Explicit engagement	Facilitator	1	0	0	0	0	0	0
Salhi et al. 2020, *Geoheritage*	Assessment of Geosites in Northern Morocco: Diversity and Richness with Potential for Socioeconomic Development	Implicit understanding	Facilitator	1	1	0	0	1	1	0
Scarlett and Riede 2019, *Geoheritage*	The Dark Geocultural Heritage of Volcanoes: Combining Cultural and Geoheritage Perspectives for Mutual Benefit	Implicit understanding	Facilitator	0	0	0	0	1	1	0
Schrodt et al. 2024, *Philosophical Transactions of the Royal Society A*	The status and future of essential geodiversity variables	Mentioned only	Facilitator	0	0	0	0	0	0	0
Selmi et al. 2022, *Sustainability*	Degradation Risk Assessment: Understanding the Impacts of Climate Change on Geoheritage	Implicit understanding	Facilitator	0	0	1	0	1	1	0
Stavi et al. 2019, *Catena*	Geodiversity effects on soil quality and geo-ecosystem functioning in drylands	Implicit understanding	Facilitator	1	1	1	0	0	0	0
Stavi et al. 2021, *Ecology and Evolution*	Positive impacts of livestock and wild ungulate routes on functioning of dryland ecosystems	Implicit understanding	Facilitator	1	1	1	0	0	0	0
Tavares et al. 2015, *Sustainability*	Community Involvement in Geoconservation: A Conceptual Approach Based on the Geoheritage of South Angola	Implicit understanding	Facilitator	0	0	0	0	1	1	0
Tavasoli et al. 2022, *Geoconservation Research*	Investigation of Geological Diversity Based on the Degree of Sensitivity and the Amount of Equilibrium and Resilience of the Geosystem (Case Study: The Eastern Kopet-Dagh Zone)	Explicit engagement	Facilitator	1	0	1	0	0	0	0
Vegas et al. 2023, *book chapter in "El Hierro Island, Active Volcanoes of the World"*	Tagoro, the Youngest Submarine Volcano in the Spanish Geoheritage Inventory: Scientific Value, Geoconservation and Opportunities for Geotourism	Implicit understanding	Facilitator	0	0	0	0	1	0	0
Vereb et al. 2020a, *Quaestiones Geographicae*	The urban geoheritage of Clermont-Ferrand: from inventory to management	Implicit understanding	Facilitator	1	0	0	0	1	1	0
Vereb et al. 2020b, *Geoheritage*	Geoheritage and Resilience of Dallol and the Northern Danakil Depression in Ethiopia	Explicit engagement	Mixed	0	0	1	1	1	0	1
Wallis et al. 2021, *Scientific Reports*	Biodiversity and ecosystem functions depend on environmental conditions and resources rather than the geodiversity of a tropical biodiversity hotspot	Mentioned only	Mixed	0	0	0	0	0	0	0

Yuliawati et al. 2024, *Journal of Ecohumanism*	Enhancing Geo-Culinary Competitiveness of MSMEs through Sustainable Marketing in Geopark West Java Indonesia	Implicit understanding	Facilitator	1	0	0	0	1	1	0
Zacharová et al. 2022, *Land*	Historical Agricultural Landforms—Central European Bio-Cultural Heritage Worthy of Attention	Mentioned only	Facilitator	1	1	1	1	1	0	0
Zeller et al. 2024, *Landscape Ecology*	Measuring ecological connectivity with ecological distance and dynamic resistant kernels	Explicit engagement	Facilitator	0	1	0	0	0	0	0
Zhu et al. 2023, *Ecological Applications*	Biodiversity conservation adaptation to climate change: Protecting the actors or the stage	Explicit engagement	Facilitator	1	1	1	0	0	0	0

Table S4. Summary of resilience definitions and theoretical frameworks in studies with explicit engagement (n=17). Definitions are summarized from original sources; see reference list for full citations.

Reference	Definition of resilience
Alberico et al. (2023)	**The ability of a system, community, or society to withstand, absorb, adapt, and recover** from hazardous events in a timely and efficient manner, including preservation and restoration of essential structures and functions through risk management (Bozza et al. 2017). Emphasizes close connection between sustainability and resilience.
Anderson et al. (2014)	"Site resilience" as the **capacity of a geophysical site to maintain species diversity and ecological function** as climate changes, operationalized as structurally intact geophysical settings that sustain diversity, maintain ecological relationships, and allow adaptive change (modified from Gunderson 2000).
Anderson et al. (2023)	"Site resilience" as the **capacity of a place to maintain species diversity and ecological function** as climate changes, operationalized through quantitative metrics combining landscape diversity (topoclimatic variability) and local connectedness.
Baitalik (2024)	Applies "Resilience Theory" to assess **adaptability of geoheritage sites** to environmental and human-induced changes, though lacks explicit foundational resilience theory citations. Framework emphasizes adaptive management through continuous assessment, planning, implementation, and evaluation cycles.
Gordon et al. (2022)	Resilience as component of adaptive capacity, defined as **the ability to absorb and recover** from disturbance, complementing resistance (ability to resist change). Applied specifically to geoheritage features' capacity to cope with environmental change without loss of favourable condition (adapted from Kittel 2013).
Knudson et al. (2018)	Climate resilience as the **ability of a place to bounce back** from environmental disturbances; resilient landscapes provide sufficient area for species movement in response to climate-induced shifts. Traces resilience thinking from Holling's (1973) ecological resilience through social-ecological systems (SES) theory.
Moradipour et al. (2024)	"Geoheritage resilience" as the **capacity of geosites to absorb disturbance or shock and remain in consistent condition** while substantially preserving heritage values, structure, and function. Draws from (socio-)ecological resilience literature (Holling 1973; Folke 2006).
Petrosino et al. (2019)	General resilience as **capacity of a system to withstand, respond to, adapt, and prepare for shocks and stresses** to emerge stronger after hardships (Folke et al. 2009). Rooted in ecology (Holling 1973) describing natural system capacity to absorb shocks and return to equilibrium. Focus on social resilience in volcanic risk contexts.
Pijet-Migoń & Migoń (2024)	Resilience development as **ability of systems, communities, and infrastructures to regain inherent characteristics** after disturbance and cataclysmic events. Distinguished from sustainability by emphasizing flexibility and adaptation to change. Traced to ecological origins (Holling 1973) with incorporation of tourism and sustainability literature.
Powell et al. (2018)	Community resilience as **the ability of community members to develop and engage resources to adapt or maintain productivity and function** when exposed to external shocks (Magis 2010; Holladay and Powell 2013; Holladay and Powell 2016). Also references social-ecological resilience as "the amount of change a system can absorb, recover from, and maintain function" (Walker et al. 2006; Walker and Salt 2012).
Roque Guerrero et al. (2020)	Landscape resilience as the **ability of an environment to withstand disturbances and reorganize itself** while subject to forces of change, ultimately maintaining essential functions, structure, identity, and mechanisms (Walker et al. 2004).

Tavasoli et al. (2022)	Resilience as the **rate at which an eco-/geosystem returns to equilibrium** after changing conditions and turbulence; the ability to rebuild, flexibility, and adaptability with recovery (Alberti 2008; Tooth 2018). Integrates geomorphological resilience theory (Phillips 2011) with ecological and thermodynamic systems concepts.
Zeller et al. (2024)	Resilience as the **ability to absorb stressors while maintaining ecological functions** (Gunderson 2000). Operationalized through ecological connectivity metrics using resistant kernels and geodiversity variables, interpreted as facilitating recovery following disturbances (McGarigal et al. 2018).
Zhu et al. (2023)	Climate resilience operationalized as aggregate score of local connectedness and landscape diversity, representing topography and elevation heterogeneity (Anderson et al. 2014). Uses The Nature Conservancy's resilience framework emphasizing **capacity to absorb disturbances and adapt to change**.
De Pascale et al. (2017)	Multiple disciplinary perspectives: materials science (property to **maintain structure or regain original shape** after deformation); social science (**capacity to resist and re-establish stability** after losses); systems level (**capacity to re-establish equilibrium** after critical events); psychological (**individuals' capacity** to show appropriate skill following adversity). Operationalized through psychological resilience questionnaire for children.
Moretti et al. (2021)	**Capacity to restore and retain long-term functions, structure, identity, and feedbacks** after shocks (Holling 1996; Walker et al. 2004). Distinguished from sustainability: sustainability focuses on maintaining conditions over long-term, while resilience focuses on recovery from shocks. Both concepts viewed as complementary for small island developing states.
Vereb et al. (2020)	The **ability of a system, community, or society exposed to hazards to resist, absorb, accommodate to, and recover** from hazard effects in a timely and efficient manner, including preservation and restoration of essential structures and functions (UNISDR 2009). Applied to visitor and geoheritage management in hazardous geothermal environment.

References

Agastya, I. B. O., E. T. Paripurno, B. Prastistho, H. Murwanto, J. D. Prasetya, and A. R. B. Nugraho. 2024. Model media learning for disaster risk reduction in the Batur UNESCO Global Geopark. *International Journal of Geoheritage and Parks* 12: 353–366. doi:10.1016/j.ijgeop.2024.04.005.

Alahuhta, J., M. Toivanen, and J. Hjort. 2019. Geodiversity–biodiversity relationship needs more empirical evidence. *Nature Ecology & Evolution* 4: 2–3. doi:10.1038/s41559-019-1051-7.

Alberico, I., G. Alessio, M. Fagnano, and P. Petrosino. 2023. The Effectiveness of Geotrails to Support Sustainable Development in the Campi Flegrei Active Volcanic Area. *Geoheritage* 15: 15. doi:10.1007/s12371-022-00778-6.

Alberti, M. 2008. *Advances in Urban Ecology*. Boston, MA: Springer US. doi:10.1007/978-0-387-75510-6.

Aleksova, B., D. Vasiljević, K. Nemeth, and I. Milevski. 2024a. Palaeovolcanic Geoheritage from Volcano Geology Perspective within Earth's Geosystems: Geoeducation of the Potential Geopark Kratovo-Zletovo (North Macedonia). *Geoheritage* 16: 54. doi:10.1007/s12371-024-00960-y.

Aleksova, B., T. Lukić, I. Milevski, D. Puhar, and S. B. Marković. 2024b. Preliminary Assessment of Geohazards' Impacts on Geodiversity in the Kratovska Reka Catchment (North Macedonia). *Geosciences* 14: 62. doi:10.3390/geosciences14030062.

Alfama, V., M. H. Henriques, and A. Barros. 2024. The Challenging Nature of Volcanic Heritage: The Fogo Island (Cabo Verde, W Africa). *Geoheritage* 16: 34. doi:10.1007/s12371-024-00939-9.

Alyaqout, A., and F. Anzah. 2025. Application of bivariate mapping to assess geodiversity and its geomorphic constraints: A case study in Kuwait. *International Journal of Geoheritage and Parks* 13: 17–30. doi:10.1016/j.ijgeop.2025.01.002.

Anderson, M. G., M. Clark, and A. O. Sheldon. 2014. Estimating Climate Resilience for Conservation across Geophysical Settings. *Conservation Biology* 28: 959–970. doi:10.1111/cobi.12272.

Anderson, M. G., P. J. Comer, P. Beier, J. J. Lawler, C. A. Schloss, S. Buttrick, C. M. Albano, and D. P. Faith. 2015. Case studies of conservation plans that incorporate geodiversity: Geodiversity in Conservation Planning. *Conservation Biology* 29: 680–691. doi:10.1111/cobi.12503.

Anderson, M. G., M. Clark, A. P. Olivero, A. R. Barnett, K. R. Hall, M. W. Cornett, M. Ahlering, M. Schindel, et al. 2023. A resilient and connected network of sites to sustain biodiversity under a changing climate. *Proceedings of the National Academy of Sciences* 120: e2204434119. doi:10.1073/pnas.2204434119.

Arias, C., B. V. W. De Vries, R. Aguilar, J. Mariño, K. Cueva, N. Manrique, B. Zavala, and A. Ancalle. 2025. Volcanic geoheritage in Arequipa, Southern Peru: Assessment of pilot geosites for geohazard resilience. *International Journal of Geoheritage and Parks* 13: 44–67. doi:10.1016/j.ijgeop.2024.11.010.

Avagyan, A., L. Sahakyan, K. Meliksetyan, A. Hovhannisyan, D. Arakelyan, G. Galoyan, H. Melik-Adamyan, T. Grigoryan, et al. 2023. The Potential for a Geohazard-Related Geopark in Armenia. *Geoheritage* 15: 133. doi:10.1007/s12371-023-00900-2.

Baitalik, A. 2024. Why Geoheritage Matters in Geoconservation Strategies: A Case Study from the Khowai Badlands in West Bengal, India. *Geoheritage* 16: 81. doi:10.1007/s12371-024-00986-2.

Basson, U., E. Argaman, H. Yizhaq, C. Xu, Z. Xu, and I. Stavi. 2023. Subsurface geodiversity determines shrub resilience vs. mortality under long-term droughts in the Israeli Negev drylands. *Frontiers in Environmental Science* 11: 1168104. doi:10.3389/fenvs.2023.1168104.

Benito, X., A. Vilmi, M. Luethje, M. L. Carrevedo, M. Lindholm, and S. C. Fritz. 2020. Spatial and Temporal Ecological Uniqueness of Andean Diatom Communities Are Correlated With Climate, Geodiversity and Long-Term Limnological Change. *Frontiers in Ecology and Evolution* 8: 260. doi:10.3389/fevo.2020.00260.

Biggs, R., M. Schlüter, D. Biggs, E. L. Bohensky, S. BurnSilver, G. Cundill, V. Dakos, T. M. Daw, et al. 2012. Toward Principles for Enhancing the Resilience of Ecosystem Services. *Annual Review of Environment and Resources* 37: 421–448. doi:10.1146/annurev-environ-051211-123836.

Biggs, R., M. Schlüter, and M. L. Schoon. 2015. *Principles for Building Resilience: Sustaining Ecosystem Services in Social-Ecological Systems*. Cambridge: Cambridge University Press.

Bozza, A., D. Asprone, and G. Manfredi. 2017. A methodological framework assessing disaster resilience of city ecosystems to enhance resource use efficiency. *International Journal of Urban Sustainable Development* 9: 136–150. doi:10.1080/19463138.2016.1244538.

Brazier, V., P. M. C. Bruneau, J. E. Gordon, and A. F. Rennie. 2012. Making Space for Nature in a Changing Climate: The Role of Geodiversity in Biodiversity Conservation. *Scottish Geographical Journal* 128: 211–233. doi:10.1080/14702541.2012.737015.

Brown, E., C. Prosser, and N. Stevenson. 2012. Geodiversity, Conservation and Climate Change: Key Principles for Adaptation. *SCOTTISH GEOGRAPHICAL JOURNAL* 128: 234–239. WOS:000311374000005. doi:10.1080/14702541.2012.725859.

Comer, P. J., R. L. Pressey, M. L. Hunter, C. A. Schloss, S. C. Buttrick, N. E. Heller, J. M. Tirpak, D. P. Faith, et al. 2015. Incorporating geodiversity into conservation decisions: Geodiversity and Conservation Decisions. *Conservation Biology* 29: 692–701. doi:10.1111/cobi.12508.

Dakhil, M. A., J. Li, B. Pandey, K. Pan, Z. Liao, O. A. Olatunji, L. Zhang, E. M. Eid, et al. 2021. Richness patterns of endemic and threatened conifers in south-west China: topographic-soil fertility explanation. *Environmental Research Letters* 16: 034017. doi:10.1088/1748-9326/abda6e.

Das, A., K. C. Nauriyal, S. P. Prizomwala, C. Borgohain, U. R. Pandey, C. Vedpathak, D. R. Kouli, N. Deori, et al. 2024. Geoheritage of Charaideo Moidams (Assam, India): a Tale of Human Resilience and Man-Environment Relationship. *Geoheritage* 16: 69. doi:10.1007/s12371-024-00975-5.

De Falco, N., R. Tal-Berger, A. Hjazin, H. Yizhaq, I. Stavi, and S. Rachmilevitch. 2021. Geodiversity impacts plant community structure in a semi-arid region. *Scientific Reports* 11: 15259. doi:10.1038/s41598-021-94698-0.

De Pascale, F., M. Bernardo, F. Muto, D. Di Matteo, and V. Dattilo. 2017. Resilience and seismic risk perception at school: a geoethical experiment in Aiello Calabro, southern Italy. *Natural Hazards* 86: 569–586. doi:10.1007/s11069-016-2696-z.

Dos Santos Pereira, L., A. M. Rodrigues, M. Do Carmo Oliveira Jorge, A. J. T. Guerra, C. A. Booth, and M. A. Fullen. 2022. Detrimental effects of tourist trails on soil system dynamics in Ubatuba Municipality, São Paulo State, Brazil. *CATENA* 216: 106431. doi:10.1016/j.catena.2022.106431.

Everard, M., and N. Quinn. 2015. Realizing the value of fluvial geomorphology. *INTERNATIONAL JOURNAL OF RIVER BASIN MANAGEMENT* 13: 487–500. WOS:000212559700007. doi:10.1080/15715124.2015.1048457.

Fassoulas, C., E. Nikolakakis, and S. Staridas. 2022. Digital Tools to Serve Geotourism and Sustainable Development at Psiloritis UNESCO Global Geopark in COVID Times and Beyond. *Geosciences* 12: 78. doi:10.3390/geosciences12020078.

Fepuleai, A., K. Németh, and T. Muliaina. 2021. Geopark Impact for the Resilience of Communities in Samoa, SW Pacific. *Geoheritage* 13: 50. doi:10.1007/s12371-021-00578-4.

Ferrer, L. M., D. A. Rodriguez, M. C. Forti, and F. Carriello. 2021. The anthropocene landscape and ecosystem services in the closure of sand mining: Paraíba do Sul River basin – Brazil. *Resources Policy* 74: 102405. doi:10.1016/j.resourpol.2021.102405.

Folke, C. 2006. Resilience: The emergence of a perspective for social-ecological systems analyses. *Global Environmental Change* 16: 253–267. doi:10.1016/j.gloenvcha.2006.04.002.

Folke, Carl, G. Kofinas, and S. Chapin. 2009. Principles of ecosystem stewardship: resilience-based natural resource management in a changing world: 1–409. doi:10.1007/978-0-387-73033-2.

Giardino, M., S. Justice, R. Olsbo, P. Balzarini, A. Magagna, C. Viani, I. Selvaggio, M. Kiuttu, et al. 2022. ERASMUS+ Strategic Partnerships between UNESCO Global Geoparks, Schools, and Research Institutions: A Window of Opportunity for Geoheritage Enhancement and Geoscience Education. *Heritage* 5: 677–701. doi:10.3390/heritage5020038.

Gill, J. L., J. L. Blois, B. Benito, S. Dobrowski, M. L. Hunter, and J. L. McGuire. 2015. A 2.5-million-year perspective on coarse-filter strategies for conserving nature's stage: Paleo perspectives on conserving nature's stage. *Conservation Biology* 29: 640–648. doi:10.1111/cobi.12504.

Gonçalves, J., E. De Castro, F. Loureiro, and P. Pereira. 2024. Assessment of forest fires impacts on geoheritage: A study in the Estrela UNESCO Global Geopark, Portugal. *International Journal of Geoheritage and Parks* 12: 580–605. doi:10.1016/j.ijgeop.2024.11.005.

Gordon, J. E. 2019. Geoconservation principles and protected area management. *International Journal of Geoheritage and Parks* 7: 199–210. doi:10.1016/j.ijgeop.2019.12.005.

Gordon, J. E. 2023. Climate Change and Geotourism: Impacts, Challenges, and Opportunities. *Tourism and Hospitality* 4: 514–538. doi:10.3390/tourhosp4040032.

Gordon, J. E., and H. F. Barron. 2013. The role of geodiversity in delivering ecosystem services and benefits in Scotland. *Scottish Journal of Geology* 49: 41–58. doi:10.1144/sjg2011-465.

Gordon, J. E., H. F. Barron, J. D. Hansom, and M. F. Thomas. 2012. Engaging with geodiversity—why it matters. *Proceedings of the Geologists' Association* 123: 1–6. doi:10.1016/j.pgeola.2011.08.002.

Gordon, J. E., R. Crofts, E. Díaz-Martínez, and K. S. Woo. 2018. Enhancing the Role of Geoconservation in Protected Area Management and Nature Conservation. *Geoheritage* 10: 191–203. doi:10.1007/s12371-017-0240-5.

Gordon, J. E., V. Brazier, J. D. Hansom, and A. Werritty. 2019. Advances in Quaternary studies and geomorphology in Scotland: implications for geoconservation. *Earth and Environmental Science Transactions of the Royal Society of Edinburgh* 110: 257–278. doi:10.1017/S1755691019000069.
Gordon, J. E., R. Crofts, M. Gray, and D. Tormey. 2021. Including geoconservation in the management of protected and conserved areas matters for all of nature and people. *International Journal of Geoheritage and Parks* 9: 323–334. doi:10.1016/j.ijgeop.2021.05.003.
Gordon, J. E., R. M. L. Wignall, V. Brazier, R. Crofts, and D. Tormey. 2022. Planning for Climate Change Impacts on Geoheritage Interests in Protected and Conserved Areas. *Geoheritage* 14: 126. doi:10.1007/s12371-022-00753-1.
Gordon, J. E., E. J. Brown, D. R. Bridgland, and V. Brazier. 2023. Valuing the Quaternary – Nature conservation and geoheritage. *Proceedings of the Geologists' Association* 134: 375–387. doi:10.1016/j.pgeola.2023.07.003.
Gray, M. 2024. Case studies associated with the 10 major geodiversity-related topics. *Philosophical Transactions of the Royal Society A: Mathematical, Physical and Engineering Sciences* 382: 20230055. doi:10.1098/rsta.2023.0055.
Gray, M., J. E. Gordon, and E. J. Brown. 2013. Geodiversity and the ecosystem approach: the contribution of geoscience in delivering integrated environmental management. *Proceedings of the Geologists' Association* 124: 659–673. doi:10.1016/j.pgeola.2013.01.003.
Guilbaud, M. N., N. I. Villalba, E. Rodríguez Osnaya, M. D. P. Ortega-Larrocea, S. Cram-Heydrich, and E. Zeno-Lira. 2024. The multiple values of urban geosites: El Arenal and Viaje a la Naturaleza parks in Mexico City. *Geofísica Internacional* 63: 1067–1085. doi:10.22201/igeof.2954436xe.2024.63.3.1767.
Guilbaud, M.-N., M. D. P. Ortega-Larrocea, S. Cram, and B. Van Wyk De Vries. 2021. Xitle Volcano Geoheritage, Mexico City: Raising Awareness of Natural Hazards and Environmental Sustainability in Active Volcanic Areas. *Geoheritage* 13: 6. doi:10.1007/s12371-020-00525-9.
Gunderson, L. H. 2000. Ecological Resilience—In Theory and Application. *Annual Review of Ecology and Systematics* 31: 425–439. doi:10.1146/annurev.ecolsys.31.1.425.
Gupta, L., and J. Dixit. 2024. Quantification of geomorphodiversity and its spatial distribution with the flood inundation areas for Assam, India. *Environmental Monitoring and Assessment* 196: 1014. doi:10.1007/s10661-024-13200-1.
Hamidova, E., A. Bosino, L. Franceschi, and M. De Amicis. 2024. Nature-Based Solution Integration to Enhance Urban Geomorphological Mapping: A Methodological Approach. *Land* 13: 467. doi:10.3390/land13040467.
Harmon, B., and H. Viles. 2013. Beyond geomorphosites: trade-offs, optimization, and networking in heritage landscapes. *Environment Systems and Decisions* 33: 272–285. doi:10.1007/s10669-013-9448-3.
Holladay, P. J., and R. B. Powell. 2013. Resident perceptions of social–ecological resilience and the sustainability of community-based tourism development in the Commonwealth of Dominica. *Journal of Sustainable Tourism* 21: 1188–1211. doi:10.1080/09669582.2013.776059.
Holladay, P. J., and R. B. Powell. 2016. Social-ecological systems, stakeholders, and sustainability: A qualitative inquiry into community-based tourism in the Commonwealth of Dominica. *Caribbean Studies* 33. Caribbean Studies: 3–28. doi:10.1353/crb.2016.0000.
Holling, C. S. 1973. Resilience and Stability of Ecological Systems. *Annual Review of Ecology and Systematics* 4: 1–23. doi:https://www.jstor.org/stable/2096802.
Holling, C. S. 1996. Engineering Resilience versus Ecological Resilience. In *Engineering Within Ecological Constraints*, 31–44. Washington, DC: The National Academies Press.
Hoyland, R. O., M. T. McHenry, and E. A. Foster. 2024. Fire and geodiversity. *International Journal of Wildland Fire* 33: WF23134. doi:10.1071/WF23134.
Kärnä, O.-M., J. Heino, T. Laamanen, J. Jyrkänkallio-Mikkola, V. Pajunen, J. Soininen, K. T. Tolonen, H. Tukiainen, et al. 2019. Does catchment geodiversity foster stream biodiversity? *Landscape Ecology* 34: 2469–2485. doi:10.1007/s10980-019-00901-z.
Kittel, T. G. F. 2013. The Vulnerability of Biodiversity to Rapid Climate Change. In *Climate Vulnerability*, 185–201. Elsevier. doi:10.1016/B978-0-12-384703-4.00437-8.
Knudson, C., K. Kay, and S. Fisher. 2018. Appraising geodiversity and cultural diversity approaches to building resilience through conservation. *Nature Climate Change* 8: 678–685. doi:10.1038/s41558-018-0188-8.
Koupatsiaris, A. A., and H. Drinia. 2024. Expanding Geoethics: Interrelations with Geoenvironmental Education and Sense of Place. *Sustainability* 16: 1819. doi:10.3390/su16051819.
Kubalíková, L., and M. Balková. 2023. Two-level assessment of threats to geodiversity and geoheritage: A case study from Hády quarries (Brno, Czech Republic). *Environmental Impact Assessment Review* 99: 107024. doi:10.1016/j.eiar.2022.107024.

Kubalíková, L., P. N. Irapta, M. Pál, Z. Zwoliński, P. Coratza, and B. Van Wyk De Vries. 2023. Visages of geodiversity and geoheritage: a multidisciplinary approach to valuing, conserving and managing abiotic nature. *Geological Society, London, Special Publications* 530: 1–12. doi:10.1144/SP530-2022-321.
Magis, K. 2010. Community Resilience: An Indicator of Social Sustainability. *Society & Natural Resources* 23: 401–416. doi:10.1080/08941920903305674.
Magness, D. R., A. L. Sesser, and T. Hammond. 2018. Using topographic geodiversity to connect conservation lands in the Central Yukon, Alaska. *Landscape Ecology* 33: 547–556. doi:10.1007/s10980-018-0617-0.
Manríquez, H., P. Mansilla, R. Figueroa-Sterquel, and A. Moreira-Muñoz. 2019. Geodiversity meets Biodiversity: a landscape approach for biogeocultural conservation and governance in Mediterranean central Chile. *eco.mont (Journal on Protected Mountain Areas Research)* 11: 43–48. doi:10.1553/eco.mont-11-1s43.
Mariani, G. S., and R. T. Melis. 2022. The Potential for Valorisation of Archaeo-geosites Through Climate Change: Exploratory Study of the Nora Site (Sardinia, Italy). *Geoheritage* 14: 118. doi:10.1007/s12371-022-00759-9.
Matthews, T. J. 2014. Integrating Geoconservation and Biodiversity Conservation: Theoretical Foundations and Conservation Recommendations in a European Union Context. *Geoheritage* 6: 57–70. doi:10.1007/s12371-013-0092-6.
McGarigal, K., B. W. Compton, E. B. Plunkett, W. V. DeLuca, J. Grand, E. Ene, and S. D. Jackson. 2018. A landscape index of ecological integrity to inform landscape conservation. *Landscape Ecology* 33: 1029–1048. doi:10.1007/s10980-018-0653-9.
Mehdipour Ghazi, J., and P. Audra. 2022. Travertine Park in Azarshahr (NW Iran): an Opportunity for Geoheritage Conservation and Diminishing Geohazards Risk. *Geoheritage* 14: 99. doi:10.1007/s12371-022-00734-4.
Miller, S. N., P. Beier, and F. Suzart De Albuquerque. 2024. A test of Conserving Nature's Stage: protecting a diversity of geophysical traits can also support a diversity of species at a landscape scale. *Philosophical Transactions of the Royal Society A: Mathematical, Physical and Engineering Sciences* 382: 20230063. doi:10.1098/rsta.2023.0063.
Moradipour, F., A. Moradi, and M. Yamani. 2024. Geoheritage Resilience Assessment for Geoconservation Planning in Lorestan Province, Iran. *Geoheritage* 16: 7. doi:10.1007/s12371-023-00909-7.
Morante-Carballo, F., M. J. Domínguez-Cuesta, N. Paz-Salas, J. Malavé-Hernández, J. Dueñas-Tovar, and P. Carrión-Mero. 2023. Evaluation of the potential of coastal cliffs as geosites for the promotion of geotourism. *Geography and Sustainability* 4: 356–371. doi:10.1016/j.geosus.2023.08.003.
Moretti, R., S. Moune, D. Jessop, C. Glynn, V. Robert, and S. Deroussi. 2021. The Basse-Terre Island of Guadeloupe (Eastern Caribbean, France) and Its Volcanic-Hydrothermal Geodiversity: A Case Study of Challenges, Perspectives, and New Paradigms for Resilience and Sustainability on Volcanic Islands. *Geosciences* 11: 454. doi:10.3390/geosciences11110454.
Morino, C., P. Coratza, and M. Soldati. 2022. Landslides, a Key Landform in the Global Geological Heritage. *Frontiers in Earth Science* 10: 864760. doi:10.3389/feart.2022.864760.
Németh, B., and K. Németh. 2023. Spatial decision-making support for geoheritage conservation in the urban and indigenous environment of the Auckland Volcanic Field, New Zealand. *Geological Society, London, Special Publications* 530: 235–256. doi:10.1144/SP530-2022-140.
Németh, B., K. Németh, J. Procter, and T. Farrelly. 2021. Geoheritage Conservation: Systematic Mapping Study for Conceptual Synthesis. *GEOHERITAGE* 13. WOS:000647060200001. doi:10.1007/s12371-021-00561-z.
Németh, K., and I. Gravis. 2022. Geoheritage and geodiversity elements of the SW Pacific: A conceptual framework. *International Journal of Geoheritage and Parks* 10: 523–545. doi:10.1016/j.ijgeop.2022.09.001.
Németh, K., I. Gravis, and B. Németh. 2021. Dilemma of Geoconservation of Monogenetic Volcanic Sites under Fast Urbanization and Infrastructure Developments with Special Relevance to the Auckland Volcanic Field, New Zealand. *SUSTAINABILITY* 13. WOS:000666391700001. doi:10.3390/su13126549.
Nemeth, K., M. Moufti, M. Ashor, A. Sowaigh, T. Hablil, K. Abdulhafez, and T. Sehli. 2023. The Vanishing Volcanic Geoheritage of a Key Scoria Cone and its Significance in Volcanic Hazard Resilience of the Active Monogenetic Volcanic Field near Al Madinah Kingdom of Saudi Arabia. *Geoconservation Research*. doi:10.30486/gcr.2023.1982893.1135.
Pelacani, S., M. Maerker, S. Tommasini, and S. Moretti. 2024. Combining biodiversity and geodiversity on landscape scale: A novel approach using rare earth elements and spatial distribution models in an agricultural Mediterranean landscape. *Ecological Indicators* 158: 111583. doi:10.1016/j.ecolind.2024.111583.
Petrosino, P., R. Iavarone, and I. Alberico. 2019. Enhancing Social Resilience Through Fruition of Geological Heritage in the Vesuvio National Park. *Geoheritage* 11: 2005–2024. doi:10.1007/s12371-019-00404-y.
Phillips, J. D. 2011. Emergence and pseudo-equilibrium in geomorphology. *Geomorphology* 132: 319–326. doi:10.1016/j.geomorph.2011.05.017.

Pijet-Migoń, E., and P. Migoń. 2024. New Geo- and Mining Heritage-Based Tourist Destinations in the Sudetes (SW Poland)—Towards More Effective Resilience of Local Communities. *Sustainability* 16: 5626. doi:10.3390/su16135626.

Powell, R. B., T. F. Green, P. J. Holladay, K. E. Krafte, M. Duda, M. T. Nguyen, J. H. Spencer, and P. Das. 2018. Examining Community Resilience to Assist in Sustainable Tourism Development Planning in Dong Van Karst Plateau Geopark, Vietnam. *Tourism Planning & Development* 15: 436–457. doi:10.1080/21568316.2017.1338202.

Rentier, E. S., C. Hoorn, and A. C. Seijmonsbergen. 2024. Lithium brine mining affects geodiversity and Sustainable Development Goals. *Renewable and Sustainable Energy Reviews* 202: 114642. doi:10.1016/j.rser.2024.114642.

Roque Guerrero, J., A. Teixeira Gomes, J. De Lollo, and L. Moschini. 2020. Mapping Potential Zones for Ecotourism Ecosystem Services as a Tool to Promote Landscape Resilience and Development in a Brazilian Municipality. *Sustainability* 12: 10345. doi:10.3390/su122410345.

Salhi, A., M. R. Alilou, S. Benabdelouahab, J. Vila-Subirós, P. Sala, T. Benabdelouahab, M. Himi, and A. Casas Ponsati. 2020. Assessment of Geosites in Northern Morocco: Diversity and Richness with Potential for Socioeconomic Development. *Geoheritage* 12: 88. doi:10.1007/s12371-020-00512-0.

Scarlett, J. P., and F. Riede. 2019. The Dark Geocultural Heritage of Volcanoes: Combining Cultural and Geoheritage Perspectives for Mutual Benefit. *Geoheritage* 11: 1705–1721. doi:10.1007/s12371-019-00381-2.

Schrodt, F., G. Vernham, J. Bailey, R. Field, J. E. Gordon, M. Gray, J. Hjort, C. Hoorn, et al. 2024. The status and future of essential geodiversity variables. *Philosophical Transactions of the Royal Society A: Mathematical, Physical and Engineering Sciences* 382: 20230052. doi:10.1098/rsta.2023.0052.

Selmi, L., T. S. Canesin, R. Gauci, P. Pereira, and P. Coratza. 2022. Degradation Risk Assessment: Understanding the Impacts of Climate Change on Geoheritage. *Sustainability* 14: 4262. doi:10.3390/su14074262.

Stavi, I., S. Rachmilevitch, and H. Yizhaq. 2019. Geodiversity effects on soil quality and geo-ecosystem functioning in drylands. *CATENA* 176: 372–380. doi:10.1016/j.catena.2019.01.037.

Stavi, I., H. Yizhaq, Y. Osem, and E. Argaman. 2021. Positive impacts of livestock and wild ungulate routes on functioning of dryland ecosystems. *ECOLOGY AND EVOLUTION* 11: 13684–13691. WOS:000697696000001. doi:10.1002/ece3.8147.

Tavares, A., M. Henriques, A. Domingos, and A. Bala. 2015. Community Involvement in Geoconservation: A Conceptual Approach Based on the Geoheritage of South Angola. *Sustainability* 7: 4893–4918. doi:10.3390/su7054893.

Tavasoli, S., A. Sepehr, and A. Ghaderi. 2022. Investigation of Geological Diversity Based on the Degree of Sensitivity and the Amount of Equilibrium and Resilience of the Geosystem (Case Study: The Eastern Kopet-Dagh Zone). *GEOCONSERVATION RESEARCH* 5: 303–320. WOS:000895759600004. doi:10.30486/GCR.2022.1965549.1113.

Tooth, S. 2018. The geomorphology of wetlands in drylands: Resilience, nonresilience, or …? *Geomorphology* 305: 33–48. doi:10.1016/j.geomorph.2017.10.017.

Tricco A.C., E. Lillie, W. Zarin, K.K. O'Brien, H. Colquhoun, D. Levac, et al. 2018. PRISMA Extension for Scoping Reviews (PRISMAScR): Checklist and Explanation. Annals of Internal Medicine 169: 467–473. doi:10.7326/M18-0850.

UNISDR. 2009. UNISDR terminology on disaster risk reduction. *UN Office for Disaster Risk Reduction,*.

Vegas, J., I. Galindo, J.-T. Vázquez, R. León, N. Sánchez, E. Martín-González, and C. Romero. 2023. Tagoro, the Youngest Submarine Volcano in the Spanish Geoheritage Inventory: Scientific Value, Geoconservation and Opportunities for Geotourism. In *El Hierro Island*, ed. P. J. González, 257–272. Active Volcanoes of the World. Cham: Springer International Publishing. doi:10.1007/978-3-031-35135-8_13.

Vereb, V., B. Van Wyk De Vries, M. Hagos, and D. Karátson. 2020a. Geoheritage and Resilience of Dallol and the Northern Danakil Depression in Ethiopia. *Geoheritage* 12: 82. doi:10.1007/s12371-020-00499-8.

Vereb, V., B. Van Wyk De Vries, M.-N. Guilbaud, and D. Karátson. 2020b. The Urban Geoheritage of Clermont-Ferrand: From Inventory to Management. *Quaestiones Geographicae* 39: 5–31. doi:10.2478/quageo-2020-0020.

Walker, B., and D. Salt. 2012. *Resilience Practice: Building Capacity to Absorb Disturbance and Maintain Function*. Washington, DC: Island Press/Center for Resource Economics. doi:10.5822/978-1-61091-231-0.

Walker, B., C. S. Holling, S. R. Carpenter, and A. P. Kinzig. 2004. Resilience, Adaptability and Transformability in Social-ecological Systems. *Ecology and Society* 9: art5. doi:10.5751/ES-00650-090205.

Walker, B. H., D. Salt, and W. Reid. 2006. *Resilience thinking: sustaining ecosystems and people in a changing world*. Washington: Island Press.

Wallis, C. I. B., Y. C. Tiede, E. Beck, K. Böhning-Gaese, R. Brandl, D. A. Donoso, C. I. Espinosa, A. Fries, et al. 2021. Biodiversity and ecosystem functions depend on environmental conditions and resources rather than the geodiversity of a tropical biodiversity hotspot. *Scientific Reports* 11: 24530. doi:10.1038/s41598-021-03488-1.

Yuliawati, A. K., R. Rofaida, B. P. Gautama, S. R. Panji, R. S. Millah, A. N. Aryanti, and M. S. D. Hadian. 2024. Enhancing Geo-Culinary Competitiveness of MSMEs through Sustainable Marketing in Geopark West Java Indonesia 3.

Zacharová, J., J. Riezner, J. Elznicová, I. Machová, K. Kubát, D. Holcová, M. Holec, J. Pacina, et al. 2022. Historical Agricultural Landforms—Central European Bio-Cultural Heritage Worthy of Attention. *Land* 11: 963. doi:10.3390/land11070963.

Zeller, K. A., B. W. Compton, S. P. Finn, and E. C. Palm. 2024. Measuring ecological connectivity with ecological distance and dynamic resistant kernels. *Landscape Ecology* 39: 95. doi:10.1007/s10980-024-01890-4.

Zhu, G., X. Giam, P. R. Armsworth, S. Cho, and M. Papeş. 2023. Biodiversity conservation adaptation to climate change: Protecting the actors or the stage. *Ecological Applications* 33: e2765. doi:10.1002/eap.2765.
